# B Vitamins and the Brain: Mechanisms, Dose and Efficacy—A Review

**DOI:** 10.3390/nu8020068

**Published:** 2016-01-28

**Authors:** David O. Kennedy

**Affiliations:** Brain, Performance and Nutrition Research Centre, Northumbria University, Newcastle-upon-Tyne NE1 8ST, UK; david.kennedy@northumbria.ac.uk; Tel.: +44-191-243-7720

**Keywords:** brain, coenzyme, vitamin, homocysteine, folate, thiamin, niacin, riboflavin, biotin, pantothenic acid

## Abstract

The B-vitamins comprise a group of eight water soluble vitamins that perform essential, closely inter-related roles in cellular functioning, acting as co-enzymes in a vast array of catabolic and anabolic enzymatic reactions. Their collective effects are particularly prevalent to numerous aspects of brain function, including energy production, DNA/RNA synthesis/repair, genomic and non-genomic methylation, and the synthesis of numerous neurochemicals and signaling molecules. However, human epidemiological and controlled trial investigations, and the resultant scientific commentary, have focused almost exclusively on the small sub-set of vitamins (B_9_/B_12_/B_6_) that are the most prominent (but not the exclusive) B-vitamins involved in homocysteine metabolism. Scant regard has been paid to the other B vitamins. This review describes the closely inter-related functions of the eight B-vitamins and marshals evidence suggesting that adequate levels of all members of this group of micronutrients are essential for optimal physiological and neurological functioning. Furthermore, evidence from human research clearly shows both that a significant proportion of the populations of developed countries suffer from deficiencies or insufficiencies in one or more of this group of vitamins, and that, in the absence of an optimal diet, administration of the entire B-vitamin group, rather than a small sub-set, at doses greatly in excess of the current governmental recommendations, would be a rational approach for preserving brain health.

## 1. Background

Surprisingly, given their pivotal physiological significance, our understanding of the role of the B group of vitamins (thiamine (B_1_), riboflavin (B_2_), niacin (B_3_), pantothenic acid (B_5_), vitamin B_6_, folate (B_9_) and vitamin B_12_) in health and brain function is limited in several respects. As an example, the major human epidemiological and controlled trial research effort in this area has concentrated almost exclusively on that small sub-set of B vitamins (folate, vitamin B_12_ and, to a lesser extent vitamin B_6_) that play the most obvious roles in homocysteine metabolism. The multifarious inter-related roles of the remaining five B vitamins have been largely overlooked. Possibly as a result of this, the many intervention studies that have involved administering just folic acid ± vitamins B_12_ and/or B_6_, have generated equivocal results. Similarly, whilst we have some knowledge of the minimum levels of each B vitamin required in order to prevent explicit deficiency related diseases, we have a poor understanding of the negative effects of levels of consumption that lie above the minimum, but under the optimal level of consumption for these vitamins. Indeed, we have no clear idea of where the optimal level of consumption may lie. The following review will therefore describe some of the closely inter-related cellular functions of the entire group of B vitamins in catabolic and anabolic metabolism; examine evidence from human studies suggesting widespread sub-optimal consumption of a number of these vitamins in developed societies, and the related case for consumption of these vitamins well in excess of governmental minimum recommendations. It will also marshal evidence from the largely equivocal human literature describing intervention with a small sub-set of B vitamins, and the more promising literature describing the effects of “multi-vitamin” treatments. Taken together, these strands of evidence suggest that supplementation with the entire B group of vitamins is a more rational approach than selecting one, two or three compounds from this sub-group of vitamins.

### What Are Vitamins?

Vitamins are a group of organic compounds which are essential for normal physiological functioning but which are not synthesised endogenously by the body and therefore have to be sequestered in small quantities from the diet. In total, humans require adequate amounts of 13 vitamins: four fat soluble vitamins (A, D, E, K) and nine water soluble vitamins, which comprise vitamin C and the eight B vitamins: thiamine (B_1_), riboflavin (B_2_), niacin (B_3_), pantothenic acid (B_5_), vitamin B_6_, folate (B_9_) and vitamin B_12_. The B vitamins themselves are not grouped on the basis of any chemical structural similarity, but rather with regards to their water solubility and the inter-related, cellular coenzyme functions that they play (see Section 2).

In terms of their origins, the B vitamins are typically synthesised by plants, with their synthesis in plant chloroplasts, mitochondria and the cytosol carefully regulated to the plant’s fluctuating requirements [1,2]. In the plant they perform the same cellular functions as the roles that they will go on to play in the animals that consume them, The exception to this is vitamin B_12_, which is synthesised by bacteria, and is typically sequestered from animal derived foods, with synthesis having taken place, for instance, in the foregut of ruminant animals [2].

Although most vitamins are derived ultimately from plants, they are often consumed indirectly from higher up the food chain in foods of animal origin, including meat, dairy and eggs; sometimes in forms that have already undergone some form of initial tailoring for bioactivity. Alternatively, the enzymatic tailoring to achieve their bioactive forms, as described in Table 1, will be undertaken endogenously.

One key point is that we, and other animals, have generally lost the ability to synthesise a clade-specific palette of vitamins during our evolution. The apparent evolutionary paradox of why an organism would benefit from losing the ability to synthesise a compound required for its survival is resolved by the fact that, during the course of evolution, vitamins have been in ubiquitous and plentiful supply within the food chain. An organism that can simply sequester its “vitamins” from the environment may therefore be at an evolutionary advantage, as the process of endogenous enzymatic *de novo* synthesis of these compounds would have entailed a disadvantageous cost in terms of energy expenditure, the need for cellular machinery, and the oxidative stress involved in metabolism [3,4]. With regards to human vitamin requirements, the clearest example of this process is the monosaccharide “vitamin C”, which is produced endogenously during normal metabolism by most other animals. The only exceptions to this are guinea pigs, bats, a few passerine birds and the anthropoidea (tarsiers, monkeys and apes, including humans). In the case of humans and our close primate relatives, our inability to synthesise vitamin C is due to a mutation in the gene for l-gulonolactone, an enzyme in the synthetic pathway of ascorbate, which was lost by our common ancestor some 35–55 million years ago [5]. Similarly, with respect to the B vitamins, one or more of the requisite genes expressing the enzymes required for the synthesis of vitamin B_6_ (pyridoxal 5′-phosphate) have been lost on several separate occasions in the branches of the animal kingdom since the divergence of vertebrates and invertebrates, leaving the majority of animals, including all mammals, unable to synthesise this compound endogenously [6]. Likewise, the ability to synthesise folate (vitamin B_9_) *de novo* was lost prior to the divergence of the animal kingdom, but with this clade retaining the synthetic pathway genes required to salvage and recycle folate from dietary sources [7].

**Table 1:** The B vitamins: nomenclature, dietary sources, coenzyme forms (roles), symptoms of deficiency, and risk factors (over and above low consumption).

Vitamin	Generally Known as	Good Dietary Sources	RDA ^1^ (mg)	UL ^2^	Principal Bioactive Coenzymes (and Principal Coenzyme Role [8])	Symptoms of Deficiency	Brain Specific Symptoms of Deficiency	Specific Risk Factors for Deficiency
B_1_	Thiamin(e)	Cereals (esp. whole grain), brown rice, green vegetables, potatoes, pasta, liver, pork, eggs	1.2/1.1	-	Thiamine pyrophosphate (Generation of leaving group potential)	Mild deficiency: general fatigue/weakness gastro-intestinal symptoms [9]. Deficiency: “Beri-beri”— Peripheral nerve damage and cardiovascular dysfunction leading to: pain, impaired sensory perception; swelling, weakness and pain in the limbs; shortness of breath, irregular heart rate, heart failure [10]	Mild deficiency: irritability, emotional disturbances, confusion, disturbed sleep, memory loss [9]. Deficiency: Wernicke-Korsakoff syndrome (neurodegeneration, within the medial thalamus and cerebellum). Ataxia, abnormal motor function and eye movement, amnesia, apathy, confabulation [10]	Alcohol abuse, obesity [9]
B_2_	Riboflavin	Dairy products, leafy vegetables, legumes, liver, kidneys, yeast, mushrooms	1.3/1.1	-	Flavoproteins: flavin adenine dinucleotide (FAD) or flavin mononucleotide (FMN) (redox reactions)	Weakness, oral pain/tenderness, burning/itching of the eyes, dermatitis, anaemia [11]	Fatigue, personality change, brain dysfunction [11]	inherited riboflavin malabsorption/utilisation (10%–15% prevalence) [12]
B_3_	Niacin	Meat, fish, whole grain cereal, legumes, mushrooms, nuts	16/14	35 mg	Nicotinamide adenine dinucleotide (NAD) and its phosphate (NADP) (redox reactions)	Pellagra: dermatitis/photo dermatitis, alopecia, muscle weakness, twitching/burning in the extremities, altered gait, diarrhoea [13]	Depression, anxiety, progressing to vertigo, memory loss, paranoia, psychotic symptoms, aggression (Pellagrous insanity) [13]	Alcohol abuse
B_5_	Pantothenic acid	Meat, whole grain cereals, broccoli	5	-	Co-enzyme A (CoA) (acyl activation and transfer)	Numbness/burning sensations in extremities, dermatitis, diarrhoea [14]	Encephalopathy, behaviour change, demyelination [14]	
B_6_	Vitamin B_6_ (referring to: pyridoxal, pyridoxamine, pyridoxine)	Meat, fish, legumes, nuts, bananas, potatoes	1.3/1.3 (1.7/1.5 >50 year)	100 mg	pyridoxal-5′-phosphate (PLP) and pyridoxamine-5′-phosphate (PMP) (Generation of leaving group potential)	Anaemia	Irritability, impaired alertness, depression, cognitive decline, dementia, autonomic dysfunction, convulsions [15]	Alcohol abuse, age-related malabsorption, contraceptive medications [16]
B_7_	Biotin	Eggs, liver, pork, leafy vegetables	30 (µg)	-	biotin (carboxylation reactions)	Seborrheic eczematous rash, tingling/burning of the extremities [17]	Depression, lethargy, hallucinations, seizures [17]	Type II diabetes, poor gluco-regulation [18]
B_9_	Folic acid/folate	Leafy vegetables, legumes, citrus fruits	400 (µg)	1000 µg	tetrahydrofolates inc. methyltetrahydrofolate *(One carbon transfer)*	megaloblastic anaemia, peripheral neuropathy ^3^, spinal cord lesions, metabolic abnormalities [19,20]	Affective disorders ^4^, behaviour changes, psychosis, cognitive impairment/decline, dementia (inc Alzheimer’s disease and vascular dementia) [19]	Common genetic polymorphisms (inc. MTHFR C667T) [21] Low Riboflavin and B12 [22]
B_12_	Vitamin B_12_ (referring to: the cobalamins)	Meat, fish and other animal products	2.4 (µg)	-	Methylcobalamin, adenosylcobalamin *(vicinal rearrangements)*			age-related malabsorption [23], vegetarians, vegans [24] Genetic polymorphisms [21]

^1^ Recommended Daily Allowance; ^2^ Upper limit—Food and Nutrition Board, Institute of Medicine, USA estimated “adequate intake” due to lack of data required to arrive at an RDA; ^3^ more prevalent for vitamin B12 deficiency; ^4^ more prevalent for folate deficiency.

Of course, an evolved need to sequester ubiquitous vitamins from food relies on your species continuing to readily obtain their vitamin requirements from their diet. Our pre-agricultural, micro-nutrient rich diet, which comprised of plant-derived vegetables, fruits and nuts, with fish and meat when available, has been superseded by our typical contemporary, high-energy, highly digestible, micronutrient-depleted diet. It has been suggested that this divergence between our evolutionary diet and our modern diet underlies the high levels of vitamin deficiencies seen in developed societies and many of the associated “lifestyle diseases” such as obesity, cardiovascular disease and dementia [25,26,27,28]. Certainly, research suggests that increased adherence to the “Mediterranean diet” (typified by high consumption of fruit, vegetables, legumes, complex rather than simple carbohydrates, olive oil, and red wine, and moderate consumption of fish and white meat), is associated with increased levels of all vitamins and minerals, including B vitamins. Conversely, increased adherence to the “Western” dietary pattern (typified by high consumption of processed meat, red meat, butter, high-fat dairy products, eggs, and refined grains and sugars) is associated with a general pattern of decreasing vitamin and mineral intake. This includes most of the B vitamins, with the notable exception of vitamin B_12_, which is particularly abundant in red meat [29,30].

## 2. Mechanisms of Action and Functions of B Vitamins

B vitamins act as coenzymes in a substantial proportion of the enzymatic processes that underpin every aspect of cellular physiological functioning. As a coenzyme the biologically active form of the vitamin binds within a protein “apoenzyme” creating a “holoenzyme”, thereby increasing the resultant enzyme’s competence in terms of the diversity of reactions that it can catalyse [8]. In this role, the B vitamins play key interacting roles in the majority of cellular functions. As an example of their ubiquity, the primary bioactive form of vitamin B_6_, pyridoxal 5′-phosphate, is an essential cofactor in the functioning of over 140 separate ubiquitous enzymes required for the synthesis, degradation, and interconversion of amino acids [15], whereas the active coenzyme form of pantothenic acid, coenzyme A (CoA), is an obligatory co-factor for approximately 4% of all mammalian enzymes [31]. Less often B vitamins also function as direct precursors for metabolic substrates; for example, CoA is also acetylated to form acetyl-CoA, an intermediate compound in both the generation of cellular energy and the synthesis of multiple bioactive compounds. Similarly, niacin is a precursor for ADP-ribose, which functions in multiple non-enzymatic cellular roles.

Overall, the plethora of functions undertaken by B vitamins can generally be subdivided into their roles in catabolic metabolism, leading to the generation of energy, and anabolic metabolism, resulting in the construction and transformation of bioactive molecules.

**Figure 1:**
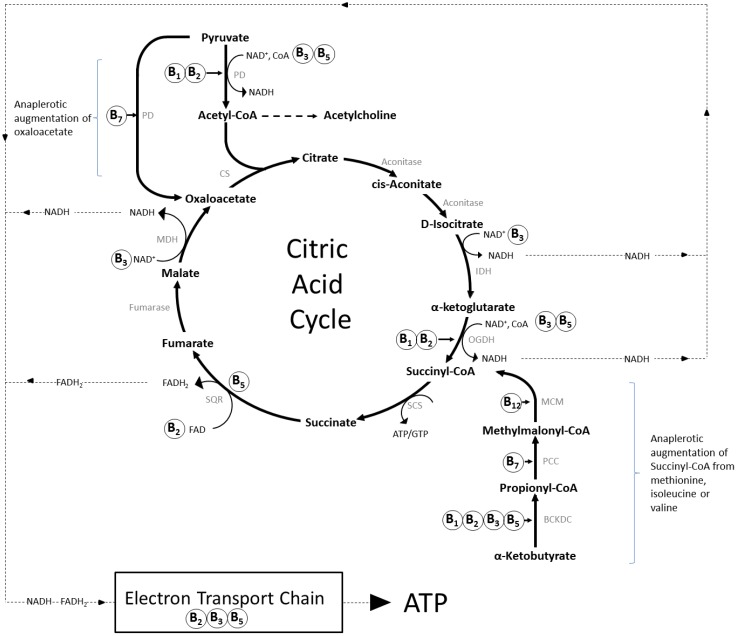
The role of B-vitamins in mitochondrial energy production. The citric acid cycle (tricarboxylic/Krebs cycle) is a series of chemical reactions that generate energy, in the form of ATP, in the mitochondria of eukaryotes. Carbohydrates, fats and proteins are first converted to acetyl-CoA, most often via pyruvate, and then undergo eight enzymatic reactions that result in the production of NADH and FADH_2_, which transfer the energy generated by the citric acid cycle to the electron transport chain. This in turn leads to the synthesis of ATP, the energy currency of cells. B vitamins contribute (as shown) to this process as co-factors/enzymes such as FAD (B_2_), NAD (B_3_) and as a component of CoA (B_5_), or Co-enzyme Q10 (B_5_). The intermediate compounds of the cycle are also sequestered as substrates for the synthesis of other compounds, including amino acids and fatty acids, and several subsequently have to be replenished by anaplerotic synthesis, taking place outside of the cycle. The most prevalent examples are the augmentation of succinyl-CoA from α-ketobutyrate generated from methionine within the methionine cycle (see Figure 2), and synthesis of oxaloacetate direct from pyruvate. Abbreviations: BCKDC, branched-chain α-ketoacid dehydrogenase complex; CS, citrate synthase; CoA, coenzyme A; FAD/FADH2, flavin adenine dinucleotide (oxidised/reduced); IDH, isocitrate dehydrogenase; NAD, nicotinamide adenine dinucleotide (+/H = oxidised/reduced); MDH, malate dehydrogenase; MCM, methylmalonyl-CoA mutase; OGDH, α-ketoglutarate dehydrogenase; PCC, propionyl-CoA Carboxylase; PC, pyruvate carboxylase; PD, pyruvate dehydrogenase; SCS, succinyl-CoA synthetase; SQR, succinate-coenzyme Q reductase.

*Catabolic energy production:* One or more of the B vitamins are involved in every aspect of the absolutely essential catabolic process of generating energy within cells [17], and deficiency in any one B vitamin will have negative consequences for this process. Of particular relevance here, the active forms of thiamine, riboflavin, niacin, and pantothenic acid are essential co-enzymes in mitochondrial aerobic respiration and cellular energy production via their direct roles in the citric acid cycle, the electron transport chain and the resultant formation of adenosine triphosphate (ATP), the cell’s energy currency. Acetyl-CoA (incorporating pantothenic acid) provides the main substrate for this cycle [9,11,14,32,33,34]. In addition, thiamine and biotin/vitamin B_12_ play unique, intersecting, essential roles in the mitochondrial metabolism of glucose [9] and fatty acids and amino acids, respectively [11], thereby contributing substrates to the citric acid cycle. The inter-related contribution of the B vitamins to the citric acid cycle and electron transport chain, the central catabolic process in mitochondria, is illustrated in Figure 1.

**Figure 2:**
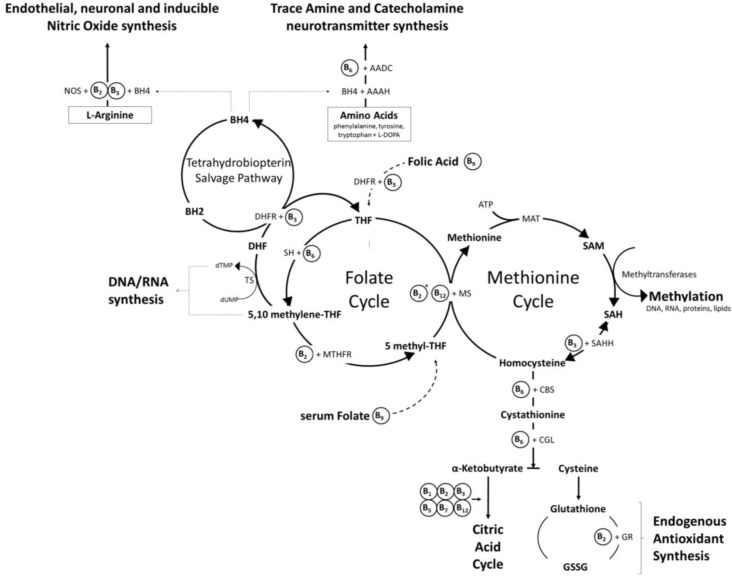
The interlinked folate and methionine cycles. Dietary folate enters the folate cycle and rotates through several enzymatic modifications which generate the one-carbon units required for the synthesis of DNA/RNA and the methyl groups required to regenerate methionine from homocysteine. The “methionine cycle” provides the methyl groups required for all genomic and non-genomic methylation reactions in the form of *S*-adenosyl methionine (SAM). These two enzymatic cycles are essential to cellular function, including via interactions with other pathways. As an example of the latter, the re-salvaging from dihydrobiopterin of tetrahydrobiopterin, an essential cofactor in trace amine and catecholamine neurotransmitter synthesis and nitric oxide production, is rate limited by provision of the enzyme dihydrofolate reductase produced by the folate cycle. * FAD (vitamin B_2_) is a cofactor for methionine synthase reductase in the recycling of the vitamin B_12_ cofactor for methionine synthase. Abbreviations: AADC, aromatic L-amino acid decarboxylase; AAAH, aromatic amino acid hydroxylases; ATP, adenosine triphosphate; BH2, dihydrobiopterin; BH4, tetrahydrobiopterin; CBS, cystathionine beta synthase; CGL, cystathionine gamma-lyase; DHFR, dihydrofolate reductase; dTMP, thymidine monophosphate; dUMP, deoxyuridine monophosphate; GR, glutathione reductase; GSSG, glutathione disulphide; MAT, methionine adenosyltransferase; MS, methionine synthase; MTHFR, methyltetrahydrofolate reductase; NOS, nitric oxide synthase; SAH, *S*-adenosylhomocysteine; SAHH, *S*-adenosylhomocysteine hydrolase; SAM, S-adenosyl methionine; SH, serine hydroxymethyltransferase; THF, tetrahydrofolate; TS, thymidylate synthase.

*Anabolic processes*: The vitamin-dependent, citric acid cycle furnishes not only energy, but also the intermediaries for the biosynthesis of numerous key compounds, including amino acids, fatty acids and pyrimidines. A number of B vitamins also play essential roles in all aspects of one-carbon metabolism [32,33,34,35], the process by which functional compounds, such as amino acids, purines, and pyrimidines, as well as methyl groups required by molecules in order for them to take part in biochemical reactions, are created within cells by the addition of single units of carbon. Of particular relevance, several B vitamin coenzymes are intrinsic contributors to two ubiquitous inter-related cellular processes: the “folate cycle”, during which tetrahydrofolate (one active form of folate) from the diet cycles through several enzymatic modifications which ultimately provide the one-carbon units required for one carbon metabolism, and the “methionine cycle” during which the amino acids methionine and homocysteine are interconverted, resulting in the synthesis of the methyl groups required for all genomic and non-genomic methylation reactions in the form of *S*-adenosyl methionine (SAM). These two enzymatic cycles are essential to cellular function, including via interactions with other pathways. As an example of the latter, the re-salvaging from dihydrobiopterin of tetrahydrobiopterin, an essential cofactor in trace amine and catecholamine neurotransmitter synthesis and nitric oxide production, is rate limited by provision of the enzyme dihydrofolate reductase produced by the folate cycle [36,37]. Similarly, the trans-sulfuration pathway that converts homocysteine to cysteine, ultimately leading to the synthesis of the potent endogenous antioxidant glutathione and the generation of substrates for the citric acid cycle, is a direct product of the methionine cycle. Whilst the roles of folate and vitamins B_6_ and B_12_ are well recognised in these intersecting cycles (see “The homocysteine hypothesis” below), the contribution of other B vitamins is rarely acknowledged. In this regard, the active form of riboflavin is a coenzyme with methyltetrahydrofolate reductase (MTHFR) in the folate cycle, and rate limits the recycling of methionine synthase in the methionine cycle [22]. Similarly, niacin, in the form of NAD, is a necessary co-factor for the enzymes dihydrofolate reductase in the folate/tetrahydrobiopterin cycles and *S*-adenosylhomocysteine hydrolase in the methionine cycle. The eventual functional products of these intersecting cellular cycles and the rate-limiting contributions made by the full range of B vitamins are illustrated in Figure 2.

Just one of the many consequences of a deficiency in any of these B vitamins (see Figure 2) is a potential hampering of the natural breakdown and recycling of homocysteine, leading to its accumulation and a number of potential, negative cellular consequences. Alongside this, the observation that homocysteine levels are increased in those suffering a range of pathologies including cardiovascular and neurodegenerative diseases has resulted in the “homocysteine hypothesis” that has driven much of the human research into the effects of B vitamins on brain function. This hypothesis will be described and discussed in more detail below.

### 2.1. Brain Specific Roles of B Vitamins

The brain is by far the most metabolically active organ in the body, representing only 2% of body weight but accounting for over 20% of the body’s total energy expenditure [38]. The B vitamins’ general metabolic functions, alongside their roles in neurochemical synthesis, may therefore be conceived as having a particular impact on brain function. Indeed, the importance of the B vitamins for brain function is illustrated by the fact that each vitamin is actively transported across the blood brain barrier and/or choroid plexus by dedicated transport mechanisms. Once in the brain, specific cellular uptake mechanisms dictate distribution, and, whilst the B vitamins all have high turnovers, ranging from 8% to 100% per day, their levels are tightly regulated by multiple homeostatic mechanisms in the brain [39,40]. This guarantees that brain concentrations remain comparatively high. For example, the concentration of methyltetrahydrofolate (the principal circulating form of folate) in the brain is four times that seen in plasma [39], whereas biotin and pantothenic acid exist in the brain at concentrations of up to 50 times that seen in plasma [41].

#### 2.1.1. Thiamine (Vitamin B_1_)

Thiamine is a coenzyme in the pentose phosphate pathway, which is a necessary step in the synthesis of fatty acids, steroids, nucleic acids and the aromatic amino acid precursors to a range of neurotransmitters and other bioactive compounds essential for brain function [9]. Thiamine plays a neuro-modulatory role in the acetylcholine neurotransmitter system, distinct from its actions as a cofactor during metabolic processes [42] and contributes to the structure and function of cellular membranes, including neurons and neuroglia [35].

#### 2.1.2. Riboflavin (Vitamin B_2_)

The two flavoprotein coenzymes derived from riboflavin, FMN and FAD are crucial rate limiting factors in most cellular enzymatic processes. As an example, they are crucial for the synthesis, conversion and recycling of niacin, folate and vitamin B_6_, and for the synthesis of all heme proteins, including hemeglobin, nitric oxide synthases, P450 enzymes, and proteins involved in electron transfer and oxygen transport and storage [11]. The flavoproteins are also co-factors in the metabolism of essential fatty acids in brain lipids [12], the absorption and utilisation of iron [43], and the regulation of thyroid hormones [11]. Dysregulation of any of these processes by riboflavin deficiency would be associated with its own broad negative consequences for brain function. Riboflavin derivatives also have direct antioxidant properties and increase endogenous antioxidant status as essential cofactors in the glutathione redox cycle [44].

#### 2.1.3. Niacin (Vitamin B_3_)

A vast array of processes and enzymes involved in every aspect of peripheral and brain cell function are dependent on niacin derived nucleotides such as nicotinamide adenine dinucleotide (NAD) and NAD phosphate (NADP). Beyond energy production, these include oxidative reactions, antioxidant protection, DNA metabolism and repair, cellular signalling events (via intracellular calcium), and the conversion of folate to its tetrahydrofolate derivative [45]. Niacin also binds agonistically at two G protein receptors, the high affinity Niacin receptor 1 (NIACR1), responsible for the skin flush associated with high intake of niacin, and the low affinity NIACR2. Niacin receptors are distributed both peripherally in immune cells and adipose tissue, and throughout the brain. Currently established roles include modulation of inflammatory cascades [46,47] and anti-atherogenic lipolysis in adipose tissue [48,49]. NIACR1 receptor populations have been shown to be down-regulated in the anterior cingulate cortex of schizophrenia sufferers [46] and upregulated in the substantia nigra of Parkinson’s disease sufferers, (a group that have low niacin levels generally) with levels correlating with poorer sleep architecture in this group [50]. A recent case study demonstrated that 250 mg niacin administration modulated peripheral immune cell NIACR1 expression and attenuated the disturbed sleep architecture associated with Parkinson’s disease [51].

#### 2.1.4. Pantothenic Acid (Vitamin B_5_)

This vitamin is a substrate for the synthesis of the ubiquitous coenzyme A (CoA). Beyond its role in oxidative metabolism, CoA contributes to the structure and function of brain cells via its involvement in the synthesis of cholesterol, amino acids, phospholipids, and fatty acids. Of particular relevance, pantothenic acid, via CoA, is also involved in the synthesis of multiple neurotransmitters and steroid hormones [14].

#### 2.1.5. Vitamin B_6_ (Pyridoxine, Pyridoxal, Pyridoxamine)

Beyond its role as a necessary cofactor in the folate cycle (see above and folate section below), the role of vitamin B_6_ in amino acid metabolism makes it a rate limiting cofactor in the synthesis of neurotransmitters such as dopamine, serotonin, γ-aminobutyric acid (GABA), noradrenaline and the hormone melatonin. The synthesis of these neurotransmitters is differentially sensitive to vitamin B_6_ levels, with even mild deficiency resulting in preferential down-regulation of GABA and serotonin synthesis, leading to the removal of inhibition of neural activity by GABA and disordered sleep, behaviour, and cardiovascular function and a loss of hypothalamus-pituitary control of hormone excretion. Vitamin B_6_ also has a direct effect on immune function and gene transcription/expression [15] and plays a role in brain glucose regulation [52]. More broadly, levels of pyridoxal-5′-phosphate are associated with increased functional indices and biomarkers of inflammation, and levels of pyridoxal-5′-phosphate are down-regulated as a function of more severe inflammation [53,54], potentially as a consequence of pyridoxal-5′-phosphate’s role either in the metabolism of tryptophan or in one-carbon metabolism [53]. This role is particularly pertinent as inflammatory processes contribute to the aetiology of numerous pathological states including dementia and cognitive decline [55].

#### 2.1.6. Biotin (Vitamin B_7_)

The brain is particularly sensitive to the delivery and metabolism of glucose. Biotin plays a key role in glucose metabolism and haemostasis, including regulation of hepatic glucose uptake, gluconeogenesis (and lipogenesis), insulin receptor transcription and pancreatic β-cell function [18]. Frank deficiency in biotin is rarely reported, although lower circulating levels of biotin have been reported in those suffering gluco-regulatory dysfunction, for instance Type II diabetes, alongside an inverse relationship between fasting plasma glucose and biotin levels [18].

#### 2.1.7. Folate (Vitamin B_9_) and Vitamin B_12_ (Cobolamin)

The functions of these two vitamins are inextricably linked due to their complementary roles in the “folate” and “methionine” cycles. Indeed, a deficiency in vitamin B_12_ results in a functional folate deficiency, as folate becomes trapped in the form of methyltetrahydrofolate [11,19]. An actual or functional folate deficiency, with an attendant reduction in purine/pyrimidine synthesis and genomic and non-genomic methylation reactions in brain tissue, leads to decreased DNA stability and repair and gene expression/transcription, which could hamper neuronal differentiation and repair, promote hippocampal atrophy, demyelination and compromise the integrity of membrane phospholipids impairing the propagation of action potentials [45]. Folate related downregulation of the synthesis of proteins and the nucleotides required for DNA/RNA synthesis, has ramifications for rapidly dividing tissue in particular, and therefore underlies the foetal developmental disorders and megaloblastic anaemia (alongside aspects of neuronal dysfunction), associated with either folate or vitamin B_12_ deficiency [11,19,45]. The efficient functioning of the folate cycle is also necessary for the synthesis and regeneration of tetrahydrobiopterin, an essential cofactor for the enzymes that convert amino acids to both monoamine neurotransmitters (serotonin, melatonin, dopamine, noradrenaline, adrenaline), and nitric oxide [56,57] (see Figure 2).

The importance of all of the B vitamins to brain function is illustrated by the neurological and psychiatric symptoms commonly associated with deficiency in any one of these eight vitamins [11,45,58,59] (see Table 1). For example, the primary symptoms of vitamin B_6_ deficiency are neurological, including depression, cognitive decline, dementia, and autonomic dysfunction [15] and vitamin B_12_ deficiency is often manifested in the form of neurological symptoms prior to the appearance of more typical haematological changes [20]. Notably, whilst about a third of those suffering folate or vitamin B_12_ deficiency present only with anaemia, a similar proportion present only with neuropsychiatric symptoms. Indeed, more than a third of psychiatric admissions have been found to be suffering deficiencies in folate or vitamin B_12_ [19].

## 3. The Homocysteine Hypothesis

No description of the mechanisms of action of the B vitamins would be complete without some consideration of the predominant mechanistic theory that has driven much of the human research in this area. The “homocysteine hypothesis” originally stemmed from the observation that increased fasting plasma levels of the potentially toxic amino acid homocysteine were an independent predictor of cardiovascular disease [60,61],with this observation subsequently extended to cognitive function [62], Alzheimer’s disease and dementia [63]. In essence, the hypothesis attributed mild to moderate increases in homocysteine levels with being a causal contributor to these disease states. Insufficiencies in several of the key vitamins involved in effectively recycling homocysteine in the methionine cycle, in particular folate, but also vitamins B_12_ and B_6_, were then implicated as the underlying cause [61]. The mechanisms by which homocysteine has been hypothesised to have these detrimental effects on brain function include its theoretical roles in increasing oxidative stress, the inhibition of methylation reactions, increased damage to DNA and dysregulation of its repair, and direct and indirect neurotoxicity leading to cell death and apoptosis. These processes are suggested to then lead to general effects such as the accumulation of beta-amyloid, hyper-phosphorylation of tau, brain tissue atrophy and compromised cerebrovascular circulation [64].

This hypothesis has been the driver not only for the majority of observational studies investigating epidemiological relationships between vitamins and cardiovascular or brain function, but also for a huge research effort that has seen a flood of clinical trials that have involved the administration of folic acid, either alone or in combination with vitamin B_12_, and less frequently, vitamin B_6_. These studies have been conducted on the basis that increasing the levels of these vitamins will reliably reduce homocysteine levels. However, the results of the intervention trials have been entirely equivocal. As an example, meta-analyses of the data from 17 trials, involving 39,107 participants [65] and 12 trials involving 47,429 participants [66] found that whereas administering folic acid ± vitamins B_12_/B_6_ reliably reduced homocysteine levels, these vitamins had no protective effect against cardiovascular or cerebrovascular disease events or all-cause mortality. The findings with regards to brain function, reviewed below, are equally equivocal. In addition, studies investigating the relationship between a common genetic polymorphism associated with higher homocysteine levels (methylenetetrahydrofolate reductase (MTHFR) 677TT) and cardiovascular disease [61] and cognitive function [67] have also been equivocal. These findings suggest that homocysteine is likely to be a simple biomarker or epiphenomenon related either to the circulating levels of the relevant vitamins or a disease related mechanism or process [61,68,69,70].

One unfortunate consequence of the “homocysteine hypothesis” is that it has effectively funneled the majority of clinical trial research in this area towards elucidating the effects of folic acid, and to a decreasing extent vitamin B_12_ followed by vitamin B_6_. The potential effects and roles of the other five B vitamins have been almost entirely ignored, despite the fact that the entire palette of B vitamins work intricately in concert. As an example, staying with the homocysteine theme, the status of folate and vitamin B_6_/B_12_ are themselves dependent on levels of riboflavin derived flavoproteins. Riboflavin is also essential for the metabolism of homocysteine as a cofactor for methylenetetrahydrofolate reductase (MTHFR) and methionine synthase reductase (MTRR) [11,12,22]. In line with this, homocysteine levels have been shown to correlate negatively with plasma riboflavin and dietary riboflavin intake [71,72], and supplementation with riboflavin has been shown to attenuate both increased homocysteine levels and blood pressure in individuals with the MTHFR 677TT polymorphism [73,74]. Although it has received even less attention than riboflavin, it is notable that niacin is also a necessary cofactor for the enzymes dihydrofolate reductase and S-adenosylhomocysteine synthase in the folate/tetrahydrobiopterin and methionine cycles, respectively, and that all of the remaining B vitamins play roles in the interlinked folate/methionine and citric acid cycles [8,11,14] (see Figure 1 and Figure 2).

The potential limitations of administering a restricted range of B vitamins are illustrated by evidence showing that approximately a third of supplementation studies to date have involved the administration of folic acid alone [65,66]. As noted above, folate and vitamin B_12_ are intimately interlinked within the folate/methionine cycles, and increasing the level of folate can mask the accrual of permanent neurological damage associated with a specific vitamin B_12_ deficiency [20]. A striking illustration of this was provided by an epidemiological study by Morris *et al.* [75] who reported that high folate status was associated with protected cognitive function, but only in those with normal vitamin B_12_ status, with this relationship reversed in participants with low vitamin B_12_ status. For this group, high folate status exacerbated the detrimental effect of vitamin B_12_ deficiency, increasing the risk of cognitive impairment and anaemia by a factor of five, compared to those with normal vitamin status. A further study also demonstrated that low vitamin B_12_ status was associated with a significantly increased decline in cognitive performance over the subsequent eight years, with this effect exacerbated in those having high levels of folate, or those taking folic acid supplements [76]. Alongside these observations it is interesting to note that in one study supplementation with folic acid also significantly increased the proportion of participants with riboflavin deficiency [72].

It is also notable, firstly, that supplementation with folic acid may not be effective in terms of regulating homocysteine: a recent study showed that folic acid supplementation reduced plasma homocysteine levels as expected, but left the more important cellular levels of homocysteine untouched, with evidence suggesting that cellular one-carbon metabolism was also disturbed [77]. Secondly, folate may affect physiological functioning via an alternative mechanism, for instance via the role the folate cycle plays in the synthesis and regeneration of tetrahydrobiopterin [57], a folate-dependent rate limiting cofactor in the enzymatic pathways to both nitric oxide and monoamine neurotransmitter synthesis [37,78,79]. This mechanism would accommodate the observation that folate increases endothelial vasodilation via a mechanism entirely unrelated to homocysteine [57,79] and would accommodate epidemiological observations of a relationship between reduced folate status and depression and disturbed cognitive function [56,78,80].

It seems reasonable to conclude, from the above and the following, that concentrating solely on one potential hypothesis as to the mechanisms of action of a small group of vitamins with multifarious complex cellular functions, at the expense of elucidating the mechanisms and effects of a broader group of inter-related vitamins, in hindsight, may not be a rational approach to research in this area.

## 4. B Vitamin Deficiencies in Developed Societies

A general assumption tends to be made that the populations of developed countries have adequate nutrition, and are therefore free from deficiencies in essential micronutrients. In order to encourage adequate nutrition, governments typically define a set of “dietary reference intakes” or similar for individual nutrients. These always include something akin to the “recommended dietary allowance”, or RDA. These government figures describe the minimum daily intake of the specific nutrient that is considered to be sufficient to meet the nutritional requirement of the majority of the healthy population. However, “meeting the requirements” in this context typically refers to simply preventing chronic, nutrition related diseases or a disease state related to a specific deficiency of that nutrient (see Table 1).

RDAs are population statistics and they therefore represent rough estimates of the average requirement of individuals within a group/population, with an adjustment for the variations in the need for the nutrient among the individuals that make up the population. However, for most micronutrients some of the information that would be required to accurately calculate the daily requirement is either unknown or incomplete, and the recommendations are therefore made on the basis of a number of assumptions and considerations that could lead to large variations in the eventual RDA [81,82]. These figures have also changed little in the last four decades, despite emerging evidence of striking individual differences in the absorption and excretion of vitamins as a consequence of a wide range of factors, including specific genetic polymorphisms, gender, ethnicity, endocrine dysfunction, thyroid function, the habitual co-consumption of medicines, drugs, alcohol and other dietary factors, obesity, overall energy consumption, vigorous exercise, and age [9,21,45,83,84,85,86]. These gaps in our knowledge question the very existence of a “normal” population [87], and suggest that RDAs are, to some extent, arbitrary figures.

Government figures also show that sizeable minorities of the populations of developed countries fail to consume even the minimum recommended quantity of any given micronutrient. As an example, Troesch *et al.* [88] presented data showing that a sizeable proportion of the populations of the US and several European countries consume less than the RDA for each of the five B vitamins that they assessed. They note that “a gap exists between vitamin intakes and requirements for a significant proportion of the population”. As a result, studies assessing the blood levels of vitamins show that small but significant proportions of the populations of developed countries have biochemical levels of each of the B vitamins that may well predispose them to deficiency related diseases. For example, UK government figures [89] show adult deficiency levels of 3% for vitamin B_12_ and 5% for folate, with these figures increasing to 5% and 12%, respectively, in the lower socio-economic portion of the population [90]. In the US, the story is similar. For instance, recent US government data [91] demonstrated that 10.5% of the entire US population were biochemically deficient in vitamin B_6_. A subsequent independent analysis that excluded the substantial minority taking supplements containing vitamin B_6_ demonstrated much higher deficiency rates of between 23% and 27% for adults, depending on age [16]. Similarly, larger proportions of “at risk” groups exhibit deficiencies in vitamin B_12_. As an example, more than 30% of a nationally representative US sample of adults over 60 years of age were below deficiency levels (<148 pmol/L) [75]. This may well be due to an age-related impairment in the absorption of the protein-bound vitamin B_12_ found in food [23], although it should be noted that deficiency levels in this vitamin are similar for vegetarians and vegans, simply due to a lack of consumption [24,92]. It has also been suggested that the available evidence suggests that the typical cut-off point defining deficiency in B_12_ is simply set too low, with negative health effects associated with reduced vitamin B_12_ extending well into “normal” levels of this vitamin [93]. Thiamine deficiency levels are also higher in the elderly, with 16%–18% deficient [94]. It is also worth noting that, whilst riboflavin deficiency levels are under-researched, biochemical deficiency is potentially widespread due to the high prevalence of an inherited restriction of riboflavin absorption/utilisation that affects 10%–15% of the world population [12].

One factor that also continues to exert an upwards pressure on deficiency levels is the paradoxical malnutrition associated with obesity. This is becoming ever more prevalent as levels of obesity continue to rise across developed nations. For instance, some 35% of the adult US population was classified as obese in 2011/12 [95]. This deficiency phenomenon is predicated largely on the basis that obesogenic diets are typically biased towards energy rich processed foods that are high in fats and simple sugars but low in micronutrients, leading to deficiencies in a range of vitamins and minerals [96]. This may be particularly prevalent for vitamins involved in, and depleted by, metabolism. For instance, thiamine plays an essential role in glucose metabolism, and between 15.5% and 29% of obese patients examined prior to bariatric surgery across a number of studies were found to be deficient. Similarly, thiamine deficiency rates have been reported to vary between 17% and 79% in patients suffering from the gluco-regulatory disease diabetes [9,18]. In a similar vein, both Type II diabetes and increased fasting plasma glucose levels have been found to be associated with lower levels of biotin [18].

Of course, an individual may not be technically deficient in a micronutrient, but may still be in the much more common state of “marginal deficiency” which will still predispose them to an increased risk of a number of more general disease states (e.g., [93,97,98]). The US government, in a recent report on micronutrient levels in the US population [91], gave their first official acknowledgement of the dangers of non-deficient but less than optimal nutritional status when the report stated that, whereas the effects of outright dietary deficiencies are well documented, “In addition, recent findings have determined that less than optimal biochemical concentrations (representing suboptimal status) have been associated with risks of adverse health effects”. Levels of marginal deficiency are, by definition, much higher than levels of outright deficiency for all of the vitamins. As an example, both Smith and Refsum [93] and Tucker *et al*. [23] noted that the neurological/psychological manifestations of vitamin B_12_ insufficiency can be evident at much higher serum levels of this vitamin than those marking deficiency. Indeed, Tucker *et al.* [23] found that whilst 9% of their sample of 3000 adults were frankly deficient in vitamin B_12_ (<148 pmol/L), over 38% had serum levels (<258 pmol/L) suggesting marginal deficiency. These figures are broadly in line with analyses of US data showing that 17.8% of all adults in the USA were marginally deficient in vitamin B_12_ using a more stringent cut-off (220 pmol/L) [96], and analyses of more recent population data showing that over 20% of the over 50 years age group in the US were marginally deficient in vitamin B_12_ between 2001 and 2006 [99]. In terms of other B vitamins, a striking 66% of the UK non-elderly adult population were at least marginally deficient in riboflavin (as assessed by the erythrocyte glutathione reductase activation test (EGRAC)) [89], with a similar figure of 54% derived in another study when a slightly more stringent EGRAC was used [72].

Taken as a whole, these figures suggest that a very sizeable proportion of the populations of developed countries are suffering deficiency or marginal deficiency in one or more B vitamins that may, at the least, dispose them to a variety of chronic diseases. Just as the minimum daily requirement of many micronutrients is simply unknown at present, the optimal level has received no attention at all. As one review paper [100] notes, even the governmental agencies responsible for defining dietary recommendations acknowledge that the benefits of micronutrient consumption may continue on a continuum well above the RDA. Clearly, common sense dictates that the optimal level of consumption of any nutrient will not merely be the level that prevents diseases related to a deficiency, or even marginal deficiency, in that nutrient. In line with this, a wealth of epidemiological evidence suggesting relationships between the increased consumption/biochemical levels of a number of vitamins, and benefits for cardiovascular function, cognitive function and decreased incidence of dementia clearly show that individuals derive additional relevant physiological benefits from consumption of micronutrients well in excess of the RDA, and biochemical levels above those denoting marginal deficiency (see [98,101]). This evidence will be summarised below.

## 5. How Much Is Enough?

As the B vitamins are water-soluble, any excess is generally excreted in urine. On the one hand, this means they are typically safe at doses much higher than the RDA, but on the other hand, they require a more consistent consumption than the fat soluble vitamins. In terms of safety, only three of the eight B-vitamins have been ascribed any upper limit for daily consumption, with the remainder considered safe at any dose [14,20]. In the case of folic acid, which is ascribed RDAs typically between 200 and 400 µg/day, the upper limit is generally set at 1000 µg/day simply on the basis that increased folate can mask the symptoms of vitamin B_12_ deficiency, allowing a hidden accumulation of permanent damage related to the latter vitamin [102]. It should also be noted that evidence suggests a potential detrimental effect of consuming high doses of folic acid, and therefore raised levels of un-metabolised folic acid, on normal folate metabolism and immune function. High folate levels may also interfere with the anti-folate medications prescribed for a number of conditions (e.g., rheumatoid arthritis, psoriasis, cancer, bacterial infections, malaria) and exert biphasic effects with regards to cancer; conferring protection at lower concentrations but increasing carcinogenesis at higher concentrations. However, to date there is no consensus as to the blood levels of folates that might cause harm [103]. The upper limit for niacin is set at 35 mg (US/Canada), with this predicated simply on its ability to cause temporary flushing of the skin at doses in excess of 100 mg, although nausea, vomiting, diarrhoea and in very rare cases liver damage have been noted following extended consumption of doses of a gram and more [8]. The final B vitamin with an ascribed upper limit is vitamin B_6_ which has an upper limit set at 100 mg/day (approximately 75 × RDA) in the US on the basis of case reports of reversible sensory neuropathy following doses in excess of 1000 mg taken for extended periods. However, it is notable that multiple clinical trials entailing consuming up to 750 mg/day of vitamin B_6_ for a number of years have demonstrated a lack of neuropathic side effects [15].

As noted above, the optimum level of any micronutrient must lie well above the RDA, and the B vitamins can generally be consumed at many times the RDA. This raises the question of how much of these vitamins should we consume? Whilst this issue is poorly understood to date, several strands of evidence suggest that increasing consumption well above the RDA should be a more effective strategy. The first strand of evidence for this comes from dose-ranging studies that have demonstrated increases in bioavailability persisting well above the RDA. For instance, Smithline *et al.* [104] demonstrated a shallow, linear dose response following single oral doses of thiamine in terms of whole blood and plasma levels up to the maximum administered dose of 1500 mg (corresponding to more than 1000 times the RDA), in healthy subjects. Similarly, one study [105] demonstrated an approximately linear dose-response in serum levels of vitamin B_12_ among adults which persisted to more than 100 µg/day of supplement use (40 × RDA), but with a plateau in levels at lower doses being evident for middle-aged and older adults. A subsequent meta-analysis [106] of the results of vitamin B_12_ supplementation studies with doses ranging from approximately 1 RDA up to 400 RDA (*i.e.*, 1000 µg) administered for between four weeks and two years, showed that for every doubling of intake above the RDA, blood levels of Vitamin B_12_ continued to increase by 11%, while methylmalonic acid levels, an indicator of deficiency, decreased by 7%. This dose response is potentially most relevant to older adults (>50 years), who typically suffer age associated malabsorption of dietary vitamin B_12_ and therefore high levels of insufficiency. Certainly, in a dose-response study, Eussen [107] found that the most effective dose for normalising vitamin B_12_ status in marginally deficient older adults was 500 µg/day (200 × RDA). It is also notable that a dose of 1 mg/day folic acid (2.5 × RDA) for 12 months was required to achieve maximal steady state erythrocyte folate concentrations in older adults [108].

In terms of potentially beneficial physiological responses to increased dosage, Eussen *et al*.’s [107] study was particularly interesting in that it also demonstrated a linear negative dose-response up to the maximum dose of 1000 µg/day Vitamin B_12_ (*i.e.*, 400 × RDA) with regards to the reductions in plasma levels of homocysteine. A clear dose response was also evident in a meta-analysis of 25 folic acid studies, with 800 µg/day (2–4 × RDA) required to achieve peak reductions in plasma homocysteine of 23%, with the addition of a median dose of 400 µg/day vitamin B_12_ (*i.e.*, 166 × RDA) associated with a further fall of 7% [109]. Interestingly, single doses of folic acid, and chronic supplementation with vitamin B_6_, folic acid and their combination, all taken at a minimum of 12 times their RDAs have all been shown to improve endothelial function in patient groups or following laboratory induced endothelial dysfunction. These effects were independent of any effect of these vitamins on homocysteine levels [110,111]. In population studies, intakes of vitamin B_6_ well in excess of the RDA, along with associated biochemical levels of pyridoxal-5′-phosphate, have also been found to be inversely related to a range of inflammatory biomarkers, with those individuals exhibiting higher levels of inflammatory biomarkers requiring several times the RDA of vitamin B_6_ merely to avoid deficiency [53,54].

With regards to riboflavin, the highest of two doses (4 mg/day, *i.e.*, 3 × RDA) administered for eight weeks to young females had the greatest effects both on riboflavin status and benefits to haematological parameters [43]. It is also notable that, whilst riboflavin has no demonstrable toxicity, the maximum daily intestinal absorption is approximately 20 times the RDA of 1.3 mg. Doses of this magnitude for up to eight weeks are also required to replenish riboflavin levels and correct enzymatic activity in the 10%–15% of the population who have an inherited restriction in their ability to absorb riboflavin [12].

Evidence (see below) also shows that “mega-doses” of biotin and niacin at between 30 and 500 times the RDA exert beneficial physiological effects, in terms of glycaemic control, insulin sensitivity, and anti-inflammatory properties. As an example, niacin, at pharmacological doses in excess of 1 g/day, has been shown to exert anti-inflammatory properties via niacin receptor interactions [47,112] and improve insulin sensitivity, reduced adipocyte size, and exert anti-atherogenic effects on lipid profiles, whilst increasing the expression of niacin receptors in adipocytes [49].

In general, epidemiological evidence suggests that the benefits of B vitamins extend well beyond the accepted biochemical cut-offs for deficiency or marginal deficiency [101] and that consuming the RDA for some B vitamins would still leave large proportions of the population at risk of insufficiency [16]. Indeed, there would seem to be little evidence for supplementing with the bare minimum requirement (RDA) given the dose-response to B vitamins in terms of bioavailability and physiological benefits.

## 6. Do B Vitamins Have an Impact on Brain Function?

Given that B vitamins are essential for every aspect of brain function, and that large proportions of the population of developed societies have less than optimal levels of vitamins, it would be expected that a relationship would be evident between vitamin consumption and mental function both in terms of epidemiological studies and controlled intervention trials. The driver for much of the research conducted to date in both of these domains has been the “homocysteine hypothesis” described above. Concentrating on this one unproven hypothesis has resulted in both observational and controlled trial research being focussed disproportionately on just three of the vitamins—folate and vitamins B_6_ and B_12_. However, the observational and the controlled trial research concentrating on these three vitamins could be seen as generating somewhat different conclusions.

### 6.1. Observational Studies

To give an idea of the size of the epidemiological research effort in this area, a review paper published in 2008 by Smith [64] summarised the relevant research published in the previous 10 years. It described, irrespective of quality, a total of 84 cross-sectional studies and 25 prospective studies that had investigated the relationship between homocysteine and/or B vitamins and brain function. Of these, 77 of the cross-sectional studies, with a total sample of more than 34,000 subjects, demonstrated a negative relationship between cognitive deficits or dementia and the status of folate or vitamins B_12_ or B_6_ and/or the opposite relationship with regard to homocysteine levels. Only seven studies incorporating ~10% of the number of subjects failed to report such relationships. Similarly, 13 prospective studies incorporating more than 7000 subjects described a relationship between baseline homocysteine and subsequent cognitive deficits measured between 2.3 and 8 years later. Similar, but less striking negative relationships between vitamin status at the outset and subsequent cognitive decline were evident in six of the 16 studies that assessed vitamin B_12_ and 10 of the 19 studies that assessed levels of folate. It was particularly noteworthy that less than 10% of the studies incorporated in the review included an assessment of vitamin B_6_, and no studies investigated the relationships pertaining to any of the remaining five B vitamins.

Since Smith’s [64] paper, a number of meta-analyses of data from the more methodologically rigorous, recently published studies have been conducted, although it is notable that these analyses applied differing methodological inclusion criteria, and almost exclusively included studies involving samples of elderly adults. These meta-analyses show a reasonably clear relationship between homocysteine levels and dementia in cross-sectional [113] and prospective studies, with high serum homocysteine at the study outset associated with a 35% increased chance of subsequently developing dementia across eight studies [114] and a 50% greater chance of suffering clinically significant cognitive decline across a further 14 studies [115]. Interestingly, at the other end of the life-span, a single study also demonstrated a positive relationship between dietary folate intake and academic achievement in adolescents [116].

In terms of circulating vitamin status, analysis of the data from 10 cross-sectional studies and one prospective study demonstrated a relationship between low folate and vitamin B_12_ and depression [117], and analysis of data from 10 cross-sectional and three cohort studies showed that that folate, but not vitamin B_12_ was associated with cognitive impairment, typically assessed with the Mini Mental State Exam (MMSE) [118]. This fits well with the findings of O’Leary *et al*. [119] who identified 35 prospective studies but found no relationship between low serum/plasma vitamin B_12_ and risk of dementia, or cognitive function. However, the authors note that the small subset of studies that included more sensitive measures of vitamin B_12_ status (such as methylmalonic acid or holotranscobalamin) demonstrated relationships in terms of dementia or cognitive function. This conclusion was in agreement with that of Doets *et al.* [120] who conducted a slightly more restricted meta-analysis. However, it contrasted with re-analysis of the data from two studies [67] that found that whereas low vitamin B_12_ was associated with cognitive impairment and dementia, and low vitamin B_6_ was associated with cognitive impairment, there was no relationship between brain function and folate, homocysteine or the MTHFR C677TT polymorphism.

Of course, each of these meta-analyses included differing collections of studies, depending on their investigational aims and inclusion/exclusion criteria, and this factor may be all important in dictating the eventual results. As an example, Lopez da Silva *et al.* [121] in a comprehensive review encompassing the relationship between a number of micronutrients and dementia, noted that only 14 out of 31 studies that they identified that had assessed folate, and only nine out of 33 studies that had assessed vitamin B_12_ actually demonstrated decreased vitamin levels in sufferers from Alzheimer’s disease. However, no studies reported the opposite relationship, and meta-analysis of the overall data confirmed the relationships. This study was interesting in two further respects. The first was that it included a meta-analysis of data from studies in which the dementia and control populations had equivalent nutrition, ruling out the confounding effects of any disease related differences in overall diet on the results. The second was that it also illustrated the extreme bias in observational studies towards investigations involving folate and Vitamin B_12_. In contrast to this voluminous body of work, only two studies included an investigation of either thiamine or vitamin B_6_, and no studies assessed the relationships between levels of the other B vitamins and any aspect of brain function.

### 6.2. Controlled Intervention Trials

#### 6.2.1. Folate, Vitamin B_12_ and Vitamin B_6_

Whilst the substantial observational literature in this area suggests a consistent relationship between aspects of brain function and folate/B_12_ and/or homocysteine, a huge research effort predicated on the hypothesis that supplementation with these vitamins should decrease homocysteine levels and thereby either improve cognitive function or attenuate cognitive decline and the risk of dementia has generated largely equivocal results. Indeed, reviews and meta-analyses published over more than a decade have provided scant evidence to support this hypothesis [122,123,124,125,126,127]. Two recent extensive meta-analyses illustrate the equivocal nature of the data. In the first of these Ford and Almeida [128] analysed the data from 19 studies involving aged participants, and found that there was no evidence that supplementation with folic acid, alone or in combination with vitamins B_12_ and B_6_, could improve or attenuate declines in cognitive function. This finding was irrespective of the cognitive status of participants at the outset, the study duration or size, or the background folate status of the study populations. More recently, Clarke *et al.* [68] meta-analysed data from 11 studies involving a total of 22,000 aged participants who received folic acid, with additional vitamin B_12_ (10 studies) and B_6_ (eight studies) and found no evidence of benefits either in terms of global cognitive function or performance in specific cognitive domains, despite a drastic reduction in homocysteine levels. However, it may be notable that this meta-analysis excluded any trials on people with cognitive impairment or dementia and therefore did not address the question of whether these B vitamins slowed cognitive decline.

Of course, these demonstrations of a lack of efficacy have elicited a counter-commentary noting that the null findings may be due to a number of methodological factors, including: the study selection; the heterogeneity or insensitivity of the cognitive tests; the good, or bad, cognitive status of the participants at the studies’ outsets; the duration of treatment; and the pooling of data obscuring the positive findings from more methodologically rigorous studies and those in sub-populations that are more likely to see benefits including those with poorer vitamin status [101,129,130,131]. Examples of the latter include positive findings in groups suffering high levels of homocysteine at the outset [132,133]. It has also been noted [129] that more consistent evidence exists for lower vitamin B_12_ status and higher homocysteine levels being associated with decreased brain volume [134,135] and increased white matter lesions [136] and for supplementation with homocysteine lowering B vitamins attenuating the rate of cerebral atrophy associated with dementia and age related cognitive impairment, particularly in those with higher homocysteine levels at the outset [137,138]. A further crumb of comfort was also provided by a recent meta-analysis [139] of 10 studies involving supplementation with folic acid, plus vitamin B_12_ (four studies) and vitamin B_6_ (three studies) and a single study of vitamin B_12_ monotherapy, and depression in sufferers of mood disorders. Whilst this analysis showed no global benefits of supplementation on depressive symptoms across studies, the subset of three studies that assessed relapse or prevention demonstrated significant benefits for B vitamin treatment. It may be relevant that these were also the three studies that administered all three B vitamins.

Interestingly, the commentary surrounding the equivocal nature of the evidence in this area has not included any reference to the predominant use of elderly participants in these studies, or whether providing an absolute maximum of three B vitamins (folate, B_6_, B_12_), simply on the basis that these will reduce levels of homocysteine, is a rational approach, given the inextricably inter-linked functions of all eight B vitamins (and the potential for deficiencies/insufficiencies in any of these vitamins).

#### 6.2.2. Thiamine, Riboflavin, Biotin, Pantothenic Acid, Niacin

Unfortunately, there is a general dearth of controlled trial research into the effects of the remaining B vitamins on brain function, or indeed any aspect of functioning in humans. Some supportive evidence does exist that shows that several of this group can modulate peripheral cardiovascular and gluco-regulatory function—and it is certainly the case that modulation of these parameters should have an impact on brain function. For instance, administration of 1.6 mg/day of riboflavin attenuated the hypertensive effect of the MTHFR 677TT genotype [140] and up to 4 mg/day led to dose-related increases in the number of circulating red blood cells and the concentration of haemoglobin [43]. Additionally, large doses (60+ × RDA) of biotin, with [141,142] or without additional chromium [143,144] have been shown to improve glycaemic control and/or insulin sensitivity in sufferers from diabetes. Similarly, both single intravenous and chronic oral mega-doses of biotin have been shown to improve lipid profiles in humans [144,145]. Finally, a meta-analysis of the data from 11 studies involving niacin supplementation confirmed that high doses (typically 1–4 g) either with or without statins reduced the incidence of cardiovascular disease and coronary heart disease events, but that this was not related to niacin’s beneficial effects on blood lipid profiles [146]. Other potential mechanisms underlying these effects include beneficial effects on inflammatory biomarkers [147] via modulation of NIACR1 receptors [112].

A single study has also assessed the direct effects of 50 mg (*i.e.*, 40 × RDA) of thiamine or placebo administered for two months to 120 young females with adequate thiamine status at the study outset. The results showed that thiamine improved mood as assessed by the Profile of Mood States, and improved attention as evinced by faster decision times in two-choice, four-choice and eight-choice reaction time tasks [148].

#### 6.2.3. Multivitamins and Brain Function

Despite clear evidence that the cellular functions of B vitamins are closely inter-related, no research to date has attempted to elucidate the effects of a full range of B vitamins with regard to any aspect of brain function (or indeed any other function). However, a growing body of research has assessed the effects of multi-vitamins/minerals which include a full range of B vitamins. Whilst the comparative contributions of the B vitamins in these treatments cannot ultimately be differentiated from those of the other vitamins and minerals in the interventions, these treatments could certainly be conceived as providing a clearer picture of the effects of “B vitamins” as a group than the research that has focussed on folic acid, often with additional vitamin B_12_ and sometimes with vitamin B_6_. This research can typically also be differentiated from that summarised above on the basis that it has typically employed samples of cognitively intact, children and non-elderly adults.

#### 6.2.4. Acute Effects of Multivitamins

Interestingly, the orthodoxy that vitamins have to be administered for an extended period of time in order to elicit any physiological effects is not based on any evidence that vitamins do not exert acute effects. Comparatively few studies have assessed the acute effects of vitamins, but from those studies that have, there is emerging evidence that vitamins have physiological and brain function effects following a single dose. For instance, single doses of a range of single vitamins, including folic acid (as well as vitamins C, E, A), administered at “mega-doses” of between five and 26 times the RDA for that micronutrient, have all been shown to increase vasodilation in groups with disease-related or experimentally induced endothelial dysfunction [149,150,151,152,153]. Acute administration of vitamin B_6_ has also been shown to elicit increased serotonin synthesis in the primate brain [154], whilst, in a placebo controlled, double blind, cross-over study in humans, the higher of two single doses of vitamin B_6_ (100 mg, 250 mg) also engendered an increase in dream salience (vividness, bizarreness, emotionality, and color) [155].

The direct acute effects of single doses of multi-vitamins (plus minerals) on brain function have also been assessed in several studies. Haskell *et al.* [156] investigated the effects of a multivitamin/mineral on cognitive function in children after a single dose (and after four and eight weeks) and found that improvements in attention task performance and in a semantic memory task were evident as early as 3 h following the first dose. Two studies have also demonstrated that a single dose of a multi-vitamin/mineral can significantly modulate regional brain activity during a task measuring focussed attention as measured with functional magnetic resonance spectroscopy (fMRI) [157], and cerebro-electrical activity during an attention task as measured by electroencephalography (EEG) [158]. In the latter study EEG changes following the multi-vitamin treatment correlated with changes in task performance. A recent study [159] also investigated the impact of two doses of multi-vitamins/minerals that differed on the basis of their water soluble vitamin content (1 RDA and 3 RDA) on cerebral blood-flow in the frontal cortex (using Near Infrared Spectroscopy) and overall energy expenditure and metabolism (using Indirect Calorimetry of exhaled gas) during difficult cognitive tasks. This study demonstrated significantly increased fat metabolism and overall energy expenditure during cognitive task performance within 2 h of consuming the higher dose (3 RDA) of water soluble vitamins, and increased cerebral blood-flow following the lower 1 RDA dose of vitamins.

#### 6.2.5. Chronic Effects of Multi-Vitamins in Children

In terms of supplementation with multi-vitamins, Benton [160], reviewed the results of studies published within the preceding decade that had assessed the effects of supplementation with multi-vitamin/minerals on children’s intelligence (IQ). All of the treatments included a full range of B vitamins, typically administered at much higher levels than the adult RDA. Benton noted evidence of improved performance in 10 out of the 13 studies, with improvements exclusively restricted to non-verbal tests of intelligence (*i.e.*, those “fluid” intelligence tasks that do not require knowledge or vocabulary and which could therefore be conceived as more closely reflecting the biological functioning of the brain). Eilander *et al.* [161] revisited the subject with a meta-analysis that included 15 multivitamin mineral studies, 12 of which had involved administration of a full range of B vitamins, with a further two of the remainder including folate and vitamins B_12_ and B_6_ alongside other vitamins. They concluded that there was evidence of a “marginal increase in fluid intelligence and academic performance in healthy schoolchildren”. Similarly, Frensham *et al.* [162] reviewed those studies from developed countries that included effect sizes and identified 10 studies that showed cognitive benefits, as opposed to four that did not. They concluded that these results show that multivitamin supplementation may engender benefits in nonverbal intelligence and in other behavioural measures.

#### 6.2.6. Chronic Effects of Multi-Vitamins in Adults

With regards multi-vitamins and adults, Kennedy and Haskell [28] identified 10 studies involving chronic multi-vitamin supplementation, almost exclusively conducted in cohorts of non-elderly adults. Across these 10 studies, all but one study reported improved psychological/cognitive functioning following supplementation, although four studies found these effects were restricted to sub-groups within their sample. In a subsequent meta-analysis of some of the cognitive data from 10 controlled trials of multi-vitamins that employed several similar memory measures, Grima *et al.* [163] found that multi-vitamin supplementation improved performance of some memory tasks, with too little data on tasks assessing other cognitive domains to arrive at a conclusion. A subsequent meta-analysis of the data from eight studies that included an assessment of the effects of multivitamins on aspects of mood and psychological state [100] found that supplementation reduced clinical ratings of perceived stress, mild psychiatric symptoms and anxiety. Of particular interest, the studies included in this analysis could be subdivided into those that administered higher (4RDA) levels of B vitamins with lower levels of other micronutrients, or lower (1RDA) levels of B vitamins with higher levels of other micronutrients. This analysis suggested that higher B vitamins with lower levels of other micronutrients engendered stronger effects, suggesting both a dose-response and that the efficacy of the products lay primarily with the B vitamin constituents. These conclusions received further support from a more recent study that also demonstrated improved mood following four weeks of supplementation with a multivitamin containing high levels of B vitamins [164]. Interestingly, several of the studies included in the reviews described above also included assessments of homocysteine levels before and after treatment, and demonstrated both that homocysteine levels were approaching levels indicating cardiovascular risk in the studies’ healthy, non-elderly samples, and also that multivitamins normalised these levels [165] including in a dose-related manner when 1RDA and 3RDA of B vitamins were administered [159].

In contrast to the benefits seen across a wide range of studies, a recent large study of long-term (12 years) multivitamin supplementation in over 5000 elderly (average 71.6 years at commencement) male retired doctors showed no cognitive effects. However, the interpretation of this study was limited by several factors: it employed a relatively crude cognitive assessment undertaken over the telephone; the participants were elderly, well-nourished, and highly educated; only one eighth of the sample received a true placebo, with three quarters of the multivitamin placebo group receiving combinations of vitamins A, C and E; and finally, the B vitamins were administered at approximately 1RDA, with the exception of vitamins B_12_ (10 × RDA) and B_6_ (2.5 × RDA). Finally, and most interestingly, whilst this study was explicitly investigating the potential for vitamins to attenuate cognitive decline in the elderly, there was no evidence of declining performance over the 12 years of the study in either the placebo or multivitamin groups [166].

It is also worth noting that a number of other recent studies have also demonstrated improved psychological or cognitive functioning following products containing multi-vitamins [167,168,169,170,171], although the interpretation of these studies with regards their vitamin content is limited by the inclusion of multiple herbal extracts at potentially psychoactive levels in the formulations. However, it may be relevant that one of these studies demonstrated a correlation between improved performance in a focussed attention (Stroop) task and changes in blood levels of vitamin B_6_ following supplementation [170].

## 7. Summary and Conclusions

The B vitamins represent a group of eight essential dietary micronutrients that work closely in concert at a cellular level and which are absolutely essential for every aspect of brain function. As water soluble nutrients, they are generally safe at levels of consumption well in excess of the recommended minimum consumption levels (possibly with the exception of folic acid, see Section 5). Indeed, bioavailability and functional data suggest that consumption of most B vitamins at levels well above dietary recommendations would be warranted.

Whilst adequate levels of all of the B vitamins should be obtainable from a healthy diet, evidence suggests that large sub-sections of the populations of developed countries are suffering deficiencies or marginal deficiencies in one or more B vitamins that will predispose them to a number of negative health consequences, including less than optimal brain function. Both epidemiological and controlled intervention trial research, driven by the predominant “homocysteine hypothesis”, have overly concentrated on the relationships with brain function, and the effects of supplementation on brain function of a narrow group of three homocysteine lowering B vitamins—folate and vitamin B_12_ and, to a lesser extent, vitamin B_6_. The potential roles and effects on brain function of the remaining five inter-related B vitamins have been largely ignored. As a consequence, consistent evidence suggests that biochemical levels of this narrow band of three vitamins, and related levels of the amino-acid homocysteine, correlate positively and negatively with brain function, respectively. However, the evidence that supplementation with one or more of these three homocysteine lowering vitamins in isolation improves brain function is entirely equivocal.

The lack of demonstrable efficacy seen in multiple meta-analyses of supplementation trials involving this small sub-group of homocysteine lowering B vitamins has often prompted a counter commentary that persists with the notion that the underlying homocysteine hypothesis is likely to be correct, suggesting rather that the methodology or focus of the individual studies or meta-analyses are incorrect, and that future research should be directed towards sub-groups of the population more likely to benefit, in trials that employ more sensitive measures (e.g., [131]). This may prove a fruitful approach, but given the inter-related cellular functions of the B vitamins, a more rational approach to research must be to investigate the effects of supplementation with the full range of B vitamins, at doses well in excess of the current governmental RDAs. There is no compelling argument for restricting this research either to a small sub-group of three B vitamins or to the elderly groups of subjects usually employed in these trials. Certainly, the smaller body of research investigating multivitamins, which has largely been undertaken in healthy children and non-elderly adults, suggests significant benefits to brain function following supplementation with multivitamin products containing a full range of B vitamins at levels well in excess of their RDAs.

It is also notable that treatments containing all of the B vitamins will inevitably reduce homocysteine (see [159,165]), and indeed, given the direct contribution of both niacin and riboflavin to the folate/methionine cycles, they should theoretically be more effective than small sub-groups of B vitamins in this regard. It is therefore difficult to conceive of any potential downsides to undertaking research with the full range of B vitamins. Of course, the luxury of being able to attribute any benefits to a single molecule and/or a single mechanism will be lost, but given the equivocal nature of the large body of evidence to date with regards to the homocysteine hypothesis, this loss would appear supportable, if not inevitable.

Naturally, the B vitamins, as a group and individually, also work intricately in concert with other vitamins, minerals and micronutrients. Whilst this topic is outside of the scope of the current review, it is noteworthy that a concerted research effort aimed at elucidating the full range of micronutrient interactions is warranted. For the moment, the foregoing suggests that research should, at a minimum, be redirected towards elucidating the potential benefits for brain function of both the acute and chronic administration of a full range of B vitamins rather than concentrating solely on the chronic effects of a small sub-group of three vitamins.

## Abbreviations

The following abbreviations are used in this manuscript:

AADC, aromatic l-amino acid decarboxylase
AAAH, aromatic amino acid hydroxylases
ATP, adenosine triphosphate
BCKDC, branched-chain α-ketoacid dehydrogenase complex
BH2, dihydrobiopterin
BH4, tetrahydrobiopterin
CBS, cystathionine beta synthase
CGL, cystathionine gamma-lyase
CoA, coenzyme A
CS, citrate synthase
DHFR, dihydrofolate reductase
dTMP, thymidine monophosphate
dUMP, deoxyuridine monophosphate
EEG, electroencephalography
EGRAC, erythrocyte glutathione reductase activation test
FAD/FADH_2_, flavin adenine dinucleotide (oxidised/reduced)
fMRI, functional magnetic resonance imaging
GABA, gamma-aminobutyric acid
GSSG, glutathione disulphide
IDH, isocitrate dehydrogenase
MAT, methionine adenosyltransferase
MDH, malate dehydrogenase
MCM, methylmalonyl-CoA mutase
MMSE, Mini Mental State Exam
MS, methionine synthase
MTHFR, methyltetrahydrofolate reductase
MTRR, methionine synthase reductase
NAD, nicotinamide adenine dinucleotide (+/H = oxidised/reduced)
NIACR, Niacin receptor
NOS, nitric oxide synthase
OGDH, α-ketoglutarate dehydrogenase
PCC, propionyl-CoA Carboxylase
PC, pyruvate carboxylase
PD, pyruvate dehydrogenase
RDA, Recommended Daily Allowance
RDI, Recommended Daily Intake
SAH, *S*-adenosylhomocysteine
SAHH, *S*-adenosylhomocysteine hydrolase
SAM, *S*-adenosyl methionine
SCS, succinyl-CoA synthetase
SH, serine hydroxymethyltransferase
SQR, succinate-coenzyme Q reductase
THF, tetrahydrofolate
TS, thymidylate synthase

## References

[1] Smith A.G., Croft M.T., Moulin M., Webb M.E. (2007). Plants need their vitamins too. Curr. Opin. Plant Biol..

[2] Kennedy D.O. (2014). Plants and the Human Brain.

[3] Banhegyi G., Braun L., Csala M., Puskas F., Mandl J. (1997). Ascorbate metabolism and its regulation in animals. Free Radic. Biol. Med..

[4] Pauling L. (1970). Evolution and the need for ascorbic acid. Proc. Natl. Acad. Sci. USA.

[5] Nishikimi M., Kawai T., Yagi K. (1992). Guinea pigs possess a highly mutated gene for l-gulono-gamma-lactone oxidase, the key enzyme for l-ascorbic acid biosynthesis missing in this species. J. Biol. Chem..

[6] Tanaka T., Tateno Y., Gojobori T. (2005). Evolution of vitamin B-6 (pyridoxine) metabolism by gain and loss of genes. Mol. Biol. Evol..

[7] Maguire F., Henriquez F.L., Leonard G., Dacks J.B., Brown M.W., Richards T.A. (2014). Complex patterns of gene fission in the eukaryotic folate biosynthesis pathway. Genome Biol. Evol..

[8] McCormick D.B., Zempleni J., Rucker R.B., McCormick D.B., Suttie J.W. (2007). Bioorganic mechanisms important to coenzyme functions. Handbook of Vitamins.

[9] Kerns J.C., Arundel C., Chawla L.S. (2015). Thiamin deficiency in people with obesity. Adv. Nutr. Int. Rev. J..

[10] Bates C.J., Zempleni J., Rucker R.B., McCormick D.B., Suttie J.W. (2007). Thiamine. Handbook of Vitamins.

[11] Rivlin R.S., Zempleni J., Rucker R.B., McCormick D.B., Suttie J.W. (2007). Riboflavin (vitamin B_2_). Handbook of Vitamins.

[12] Sinigaglia-Coimbra R., Lopes A.C., Coimbra C.G. (2011). Riboflavin deficiency, brain function, and health. Handbook of Behavior, Food and Nutrition.

[13] Kirkland J.B., Zempleni J., Rucker R.B., McCormick D.B., Suttie J.W. (2007). Niacin. Handbook of Vitamins.

[14] Rucker R.B., Bauerly K., Zempleni J., Suttie J.W., Gregory J.F., Stover P.J. (2013). Pantothenic acid. Handbook of Vitamins.

[15] Dakshinamurti S., Dakshinamurti K., Zempleni J., Suttie J.W., Gregory J.F., Stover P.J. (2013). Vitamin b_6_. Handbook of Vitamins.

[16] Morris M.S., Picciano M.F., Jacques P.F., Selhub J. (2008). Plasma pyridoxal 5′-phosphate in the us population: The national health and nutrition examination survey, 2003–2004. Am. J. Clin. Nutr..

[17] Mock D.M., Zempleni J., Rucker R.B., McCormick D.B., Suttie J.W. (2007). Biotin. Handbook of Vitamins.

[18] Via M. (2012). The malnutrition of obesity: Micronutrient deficiencies that promote diabetes. ISRN Endocrinol..

[19] Reynolds E. (2006). Vitamin B12, folic acid, and the nervous system. Lancet Neurol..

[20] Green R., Miller J., Zempleni J., Rucker R.B., McCormick D.B., Suttie J.W. (2007). Vitamin B_12_. Handbook of Vitamins.

[21] Mitchell E.S., Conus N., Kaput J. (2014). B vitamin polymorphisms and behavior: Evidence of associations with neurodevelopment, depression, schizophrenia, bipolar disorder and cognitive decline. Neurosci. Biobehav. Rev..

[22] García-Minguillán C.J., Fernandez-Ballart J.D., Ceruelo S., Ríos L., Bueno O., Berrocal-Zaragoza M.I., Molloy A.M., Ueland P.M., Meyer K., Murphy M.M. (2014). Riboflavin status modifies the effects of methylenetetrahydrofolate reductase (MTHFR) and methionine synthase reductase (MTRR) polymorphisms on homocysteine. Genes Nutr..

[23] Tucker K.L., Rich S., Rosenberg I., Jacques P., Dallal G., Wilson P.W., Selhub J. (2000). Plasma vitamin B-12 concentrations relate to intake source in the framingham offspring study. Am. J. Clin. Nutr..

[24] Pawlak R., Parrott S.J., Raj S., Cullum-Dugan D., Lucus D. (2013). How prevalent is vitamin B12 deficiency among vegetarians?. Nutr. Rev..

[25] Cordain L., Eaton S.B., Sebastian A., Mann N., Lindeberg S., Watkins B.A., O’Keefe J.H., Brand-Miller J. (2005). Origins and evolution of the western diet: Health implications for the 21st century. Am. J. Clin. Nutr..

[26] Benzie I.F.F. (2003). Evolution of dietary antioxidants. Comp. Biochem. Physiol. A Mol. Integr. Physiol..

[27] Milton K. (2000). Back to basics: Why foods of wild primates have relevance for modern human health. Nutrition.

[28] Kennedy D.O., Haskell C.F. (2011). Vitamins and cognition: What is the evidence?. Drugs.

[29] Serra-Majem L., Bes-Rastrollo M., Román-Vinas B., Pfrimer K., Sánchez-Villegas A., Martínez-González M.A. (2009). Dietary patterns and nutritional adequacy in a mediterranean country. Br. J. Nutr..

[30] Castro-Quezada I., Román-Viñas B., Serra-Majem L. (2014). The mediterranean diet and nutritional adequacy: A review. Nutrients.

[31] Daugherty M., Polanuyer B., Farrell M., Scholle M., Lykidis A., de Crécy-Lagard V., Osterman A. (2002). Complete reconstitution of the human coenzyme a biosynthetic pathway via comparative genomics. J. Biol. Chem..

[32] Huskisson E., Maggini S., Ruf M. (2007). The role of vitamins and minerals in energy metabolism and well-being. J. Int. Med. Res..

[33] Depeint F., Bruce W.R., Shangari N., Mehta R., O’Brien P.J. (2006). Mitochondrial function and toxicity: Role of b vitamins on the one-carbon transfer pathways. Chem. Biol. Interact..

[34] Depeint F., Bruce W.R., Shangari N., Mehta R., O’Brien P.J. (2006). Mitochondrial function and toxicity: Role of the b vitamin family on mitochondrial energy metabolism. Chem. Biol. Interact..

[35] Ba A. (2008). Metabolic and structural role of thiamine in nervous tissues. Cell. Mol. Neurobiol..

[36] Crabtree M.J., Tatham A.L., Hale A.B., Alp N.J., Channon K.M. (2009). Critical role for tetrahydrobiopterin recycling by dihydrofolate reductase in regulation of endothelial nitric-oxide synthase coupling relative importance of the de novo biopterin synthesis *versus* salvage pathways. J. Biol. Chem..

[37] Bendall J.K., Douglas G., McNeill E., Channon K.M., Crabtree M.J. (2014). Tetrahydrobiopterin in cardiovascular health and disease. Antioxid. Redox Signal..

[38] Raichle M.E. (2010). Two views of brain function. Trends Cogn. Sci..

[39] Spector R. (2014). Vitamin transport diseases of brain: Focus on folates, thiamine and riboflavin. Brain Disord. Ther..

[40] Spector R., Johanson C.E. (2007). Vitamin transport and homeostasis in mammalian brain: Focus on vitamins B and E. J. Neurochem..

[41] Uchida Y., Ito K., Ohtsuki S., Kubo Y., Suzuki T., Terasaki T. (2015). Major involvement of na^+^-dependent multivitamin transporter (SLC5A6/SMVT) in uptake of biotin and pantothenic acid by human brain capillary endothelial cells. J. Neurochem..

[42] Hirsch J.A., Parrott J. (2012). New considerations on the neuromodulatory role of thiamine. Pharmacology.

[43] Powers H.J., Hill M.H., Mushtaq S., Dainty J.R., Majsak-Newman G., Williams E.A. (2011). Correcting a marginal riboflavin deficiency improves hematologic status in young women in the united kingdom (ribofem). Am. J. Clin. Nutr..

[44] Ashoori M., Saedisomeolia A. (2014). Riboflavin (vitamin B2) and oxidative stress: A review. Br. J. Nutr..

[45] Bailey L.B., Zempleni J., Rucker R.B., McCormick D.B., Suttie J.W. (2007). Folic acid. Handbook of Vitamins.

[46] Miller C.L., Dulay J.R. (2008). The high-affinity niacin receptor HM74A is decreased in the anterior cingulate cortex of individuals with schizophrenia. Brain Res. Bull..

[47] Digby J.E., McNeill E., Dyar O.J., Lam V., Greaves D.R., Choudhury R.P. (2010). Anti-inflammatory effects of nicotinic acid in adipocytes demonstrated by suppression of fractalkine, rantes, and mcp-1 and upregulation of adiponectin. Atherosclerosis.

[48] Zhang Y., Schmidt R.J., Foxworthy P., Emkey R., Oler J.K., Large T.H., Wang H., Su E.W., Mosior M.K., Eacho P.I. (2005). Niacin mediates lipolysis in adipose tissue through its g-protein coupled receptor HM74A. Biochem. Biophys. Res. Commun..

[49] Linke A., Sonnabend M., Fasshauer M., Höllriegel R., Schuler G., Niebauer J., Stumvoll M., Blüher M. (2009). Effects of extended-release niacin on lipid profile and adipocyte biology in patients with impaired glucose tolerance. Atherosclerosis.

[50] Wakade C., Chong R., Bradley E., Thomas B., Morgan J. (2014). Upregulation of GPR109A in parkinson’s disease. PLoS ONE.

[51] Wakade C., Chong R., Bradley E., Morgan J.C. (2015). Low-dose niacin supplementation modulates GPR109A, niacin index and ameliorates parkinson’s disease symptoms without side effects. Clin. Case Rep..

[52] Anitha M., Abraham P.M., Paulose C.S. (2012). Striatal dopamine receptors modulate the expression of insulin receptor, Igf-1 and Glut-3 in diabetic rats: Effect of pyridoxine treatment. Eur. J. Pharmacol..

[53] Sakakeeny L., Roubenoff R., Obin M., Fontes J.D., Benjamin E.J., Bujanover Y., Jacques P.F., Selhub J. (2012). Plasma pyridoxal-5-phosphate is inversely associated with systemic markers of inflammation in a population of us adults. J. Nutr..

[54] Morris M.S., Sakakeeny L., Jacques P.F., Picciano M.F., Selhub J. (2010). Vitamin B-6 intake is inversely related to, and the requirement is affected by, inflammation status. J. Nutr..

[55] Tracy R. (2003). Emerging relationships of inflammation, cardiovascular disease and chronic diseases of aging. Int. J. Obes..

[56] Stahl S.M. (2008). L-methylfolate: A vitamin for your monoamines. J. Clin. Psychiatry.

[57] Moat S.J., Clarke Z.L., Madhavan A.K., Lewis M.J., Lang D. (2006). Folic acid reverses endothelial dysfunction induced by inhibition of tetrahydrobiopterin biosynthesis. Eur. J. Pharmacol..

[58] Sturman J.A., Rivlin R.S. (1975). Pathogenesis of brain dysfunction in deficiency of thiamine, riboflavin, pantothenic acid, or vitamin B6. Biology of Brain Dysfunction.

[59] Thomson A.D., Marshall E.J. (2006). The natural history and pathophysiology of wernicke’s encephalopathy and korsakoff’s psychosis. Alcohol Alcohol..

[60] Collaboration H.S. (2002). Homocysteine and risk of ischemic heart disease and stroke: A meta-analysis. JAMA.

[61] Smulders Y.M., Blom H.J. (2011). The homocysteine controversy. J. Inherit. Metab. Dis..

[62] Lehmann M., Gottfries C., Regland B. (1999). Identification of cognitive impairment in the elderly: Homocysteine is an early marker. Dement. Geriatr. Cogn. Disord..

[63] Seshadri S., Beiser A., Selhub J., Jacques P.F., Rosenberg I.H., D’Agostino R.B., Wilson P.W., Wolf P.A. (2002). Plasma homocysteine as a risk factor for dementia and alzheimer’s disease. N. Engl. J. Med..

[64] Smith A.D. (2008). The worldwide challenge of the dementias: A role for b vitamins and homocysteine?. Food Nutr. Bull..

[65] Mei W., Rong Y., Jinming L., Yongjun L., Hui Z. (2010). Effect of homocysteine interventions on the risk of cardiocerebrovascular events: A meta-analysis of randomised controlled trials. Int. J. Clin. Pract..

[66] Martí-Carvajal A.J., Solà I., Lathyris D. (2015). Homocysteine-lowering interventions for preventing cardiovascular events. Cochrane Datebase Syst. Rev..

[67] Moorthy D., Peter I., Scott T.M., Parnell L.D., Lai C.-Q., Crott J.W., Ordovás J.M., Selhub J., Griffith J., Rosenberg I.H. (2012). Status of vitamins B-12 and B-6 but not of folate, homocysteine, and the methylenetetrahydrofolate reductase C677T polymorphism are associated with impaired cognition and depression in adults. J. Nutr..

[68] Clarke R., Bennett D., Parish S., Lewington S., Skeaff M., Eussen S.J., Lewerin C., Stott D.J., Armitage J., Hankey G.J. (2014). Effects of homocysteine lowering with b vitamins on cognitive aging: Meta-analysis of 11 trials with cognitive data on 22,000 individuals. Am. J. Clin. Nutr..

[69] Nilsson K., Gustafson L., Hultberg B. (2013). Elevated plasma homocysteine level in vascular dementia reflects the vascular disease process. Dement. Geriatr. Cogn. Disord. Extra.

[70] Luft F.C. (2015). Fitting homocysteine to disease models, as well as adjusting the models to the disease. J. Mol. Med..

[71] Ganji V., Kafai M.R. (2004). Frequent consumption of milk, yogurt, cold breakfast cereals, peppers, and cruciferous vegetables and intakes of dietary folate and riboflavin but not vitamins B-12 and B-6 are inversely associated with serum total homocysteine concentrations in the us population. Am. J. Clin. Nutr..

[72] Moat S.J., Ashfield-Watt P.A., Powers H.J., Newcombe R.G., McDowell I.F. (2003). Effect of riboflavin status on the homocysteine-lowering effect of folate in relation to the MTHFR (C677T) genotype. Clin. Chem..

[73] McNulty H., le Roy C.D., Strain J., Dunne A., Ward M., Molloy A.M., McAnena L.B., Hughes J.P., Hannon-Fletcher M., Scott J.M. (2006). Riboflavin lowers homocysteine in individuals homozygous for the MTHFR 677C -> T polymorphism. Circulation.

[74] Horigan G., McNulty H., Ward M., Strain J., Purvis J., Scott J.M. (2010). Riboflavin lowers blood pressure in cardiovascular disease patients homozygous for the 677C -> T polymorphism in mthfr. J. Hypertens..

[75] Morris M.S., Jacques P.F., Rosenberg I.H., Selhub J. (2007). Folate and vitamin B-12 status in relation to anemia, macrocytosis, and cognitive impairment in older americans in the age of folic acid fortification. Am. J. Clin. Nutr..

[76] Morris M.S., Selhub J., Jacques P.F. (2012). Vitamin B-12 and folate status in relation to decline in scores on the mini-mental state examination in the framingham heart study. J. Am. Geriatr. Soc..

[77] Smith D.E., Hornstra J.M., Kok R.M., Blom H.J., Smulders Y.M. (2013). Folic acid supplementation does not reduce intracellular homocysteine, and may disturb intracellular one-carbon metabolism. Clin. Chem. Lab. Med..

[78] Araújo J.R., Martel F., Borges N., Araújo J.M., Keating E. (2015). Folates and aging: Role in mild cognitive impairment, dementia and depression. Ageing Res. Rev..

[79] Zhang M., Wen J., Wang X., Xiao C. (2014). High-dose folic acid improves endothelial function by increasing tetrahydrobiopterin and decreasing homocysteine levels. Mol. Med. Rep..

[80] Papakostas G.I., Shelton R.C., Zajecka J.M., Etemad B., Rickels K., Clain A., Baer L., Dalton E.D., Sacco G.R., Schoenfeld D. (2014). l-methylfolate as adjunctive therapy for ssri-resistant major depression: Results of two randomized, double-blind, parallel-sequential trials. Am. J. Psychiatry.

[81] Young V.R. (1996). Evidence for a recommended dietary allowance for vitamin C from pharmacokinetics: A comment and analysis. Proc. Natl. Acad. Sci. USA.

[82] Levine M., Conry-Cantilena C., Wang Y., Welch R.W., Washko P.W., Dhariwal K.R., Park J.B., Lazarev A., Graumlich J.F., King J. (1996). Vitamin C pharmacokinetics in healthy volunteers: Evidence for a recommended dietary allowance. Proc. Natl. Acad. Sci. USA.

[83] Caudill M.A. (2009). Folate bioavailability: Implications for establishing dietary recommendations and optimizing status. Am. J. Clin. Nutr..

[84] Kauwell G.P.A., Wilsky C.E., Cerda J.J., Herrlinger-Garcia K., Hutson A.D., Theriaque D.W., Boddie A., Rampersaud G.C., Bailey L.B. (2000). Methylenetetrahydrofolate reductase mutation (677C -> T) negatively influences plasma homocysteine response to marginal folate intake in elderly women. Metab. Clin. Exp..

[85] Shibata K., Fukuwatari T., Ohta M., Okamoto H., Watanabe T., Fukui T., Nishimuta M., Totani M., Kimura M., Ohishi N. (2005). Values of water-soluble vitamins in blood and urine of japanese young men and women consuming a semi-purified diet based on the Japanese dietary reference intakes. J. Nutr. Sci. Vitaminol..

[86] Shibata K., Fukuwatari T., Watanabe T., Nishimuta M. (2009). Intra- and inter-individual variations of blood and urinary water-soluble vitamins in japanese young adults consuming a semi-purified diet for 7 days. J. Nutr. Sci. Vitaminol..

[87] Challem J.J. (1999). Toward a new definition of essential nutrients: Is it now time for a third “vitamin” paradigm?. Med. Hypotheses.

[88] Troesch B., Hoeft B., McBurney M., Eggersdorfer M., Weber P. (2012). Dietary surveys indicate vitamin intakes below recommendations are common in representative western countries. Br. J. Nutr..

[89] Ruston D., Hoare J., Henderson L., Gregory J., Bates C., Prentice A., Birch M., Swan G., Farron M. (2004). National Diet and Nutrition Survey: Adults Aged 19–64 Years. Volume 4: Nutritional Status (Anthropometry and Blood Analytes), Blood Pressure and Physical Activity.

[90] Nelson M., Erens B., Bates B., Church S., Boshier T. (2007). Low Income Diet and Nutrition Survey.

[91] CDC (2012). Second National Report on Biochemical Indicators of Diet and Nutrition in the US Population.

[92] Aparicio-Ugarriza R., Palacios G., Alder M., González-Gross M. (2014). A review of the cut-off points for the diagnosis of vitamin B12 deficiency in the general population. Clin. Chem. Lab. Med. (CCLM).

[93] Smith A.D., Refsum H. (2012). Do we need to reconsider the desirable blood level of vitamin B12?. J. Intern. Med..

[94] Wilkinson T.J., Hanger H.C., Elmslie J., George P.M., Sainsbury R. (1997). The response to treatment of subclinical thiamine deficiency in the elderly. Am. J. Clin. Nutr..

[95] Ogden C.L., Carroll M.D., Kit B.K., Flegal K.M. (2014). Prevalence of childhood and adult obesity in the united states, 2011–2012. JAMA.

[96] Kimmons J.E., Blanck H.M., Tohill B.C., Zhang J., Khan L.K. (2006). Associations between body mass index and the prevalence of low micronutrient levels among US adults. Medscape Gen. Med..

[97] Lotto V., Choi S.-W., Friso S. (2011). Vitamin b6: A challenging link between nutrition and inflammation in cvd. Br. J. Nutr..

[98] Carr A.C., Frei B. (1999). Toward a new recommended dietary allowance for vitamin c based on antioxidant and health effects in humans. Am. J. Clin. Nutr..

[99] Qi Y.P., Do A.N., Hamner H.C., Pfeiffer C.M., Berry R.J. (2014). The prevalence of low serum vitamin B-12 status in the absence of anemia or macrocytosis did not increase among older us adults after mandatory folic acid fortification. J. Nutr..

[100] Long S.-J., Benton D. (2013). Effects of vitamin and mineral supplementation on stress, mild psychiatric symptoms, and mood in nonclinical samples: A meta-analysis. Psychosom. Med..

[101] Morris M.S. (2012). The role of B vitamins in preventing and treating cognitive impairment and decline. Adv. Nutr. Int. Rev. J..

[102] Food and Nutrition Board, Institute of Medicine (2000). Dietary Reference Intakes for Thiamin, Riboflavin, Niacin, Vitamin B6, Folate, Vitamin B12, Pantothenic Acid, Biotin and Choline.

[103] Smith A.D., Kim Y.-I., Refsum H. (2008). Is folic acid good for everyone?. Am. J. Clin. Nutr..

[104] Smithline H.A., Donnino M., Greenblatt D.J. (2012). Pharmacokinetics of high-dose oral thiamine hydrochloride in healthy subjects. BMC Pharmacol. Toxicol..

[105] MacFarlane A.J., Shi Y., Greene-Finestone L.S. (2014). High-dose compared with low-dose vitamin B-12 supplement use is not associated with higher vitamin B-12 status in children, adolescents, and older adults. J. Nutr..

[106] Dullemeijer C., Souverein O.W., Doets E.L., van der Voet H., van Wijngaarden J.P., de Boer W.J., Plada M., Dhonukshe-Rutten R.A., In’t Veld P.H., Cavelaars A.E. (2013). Systematic review with dose-response meta-analyses between vitamin B-12 intake and european micronutrient recommendations aligned’s prioritized biomarkers of vitamin B-12 including randomized controlled trials and observational studies in adults and elderly persons. Am. J. Clin. Nutr..

[107] Eussen S.J., de Groot L.C., Clarke R., Schneede J., Ueland P.M., Hoefnagels W.H., van Staveren W.A. (2005). Oral cyanocobalamin supplementation in older people with vitamin B12 deficiency: A dose-finding trial. Arch. Intern. Med..

[108] Bradbury K.E., Williams S.M., Green T.J., McMahon J.A., Mann J.I., Knight R.G., Skeaff C.M. (2012). Differences in erythrocyte folate concentrations in older adults reached steady-state within one year in a two-year, controlled, 1 mg/d folate supplementation trial. J. Nutr..

[109] Homocysteine Lowering Trialists’Collaboration (2005). Dose-dependent effects of folic acid on blood concentrations of homocysteine: A meta-analysis of the randomized trials. Am. J. Clin. Nutr..

[110] Miner S.E.S., Cole D.E.C., Evrovski J., Forrest Q., Hutchison S., Holmes K., Ross H.J. (2001). Pyridoxine improves endothelial function in cardiac transplant recipients. J. Heart Lung Transplant..

[111] Moat S.J., Lang D., McDowell I.F., Clarke Z.L., Madhavan A.K., Lewis M.J., Goodfellow J. (2004). Folate, homocysteine, endothelial function and cardiovascular disease. J. Nutr. Biochem..

[112] Wakade C., Chong R. (2014). A novel treatment target for parkinson’s disease. J. Neurol. Sci..

[113] Ho R.C., Cheung M.W., Fu E., Win H.H., Zaw M.H., Ng A., Mak A. (2011). Is high homocysteine level a risk factor for cognitive decline in elderly? A systematic review, meta-analysis, and meta-regression. Am. J. Geriatr. Psychiatry.

[114] Wald D.S., Kasturiratne A., Simmonds M. (2011). Serum homocysteine and dementia: Meta-analysis of eight cohort studies including 8669 participants. Alzheimer’s Dement..

[115] Nie T., Lu T., Xie L., Huang P., Lu Y., Jiang M. (2014). Hyperhomocysteinemia and risk of cognitive decline: A meta-analysis of prospective cohort studies. Eur. Neurol..

[116] Nilsson T.K., Yngve A., Böttiger A.K., Hurtig-Wennlöf A., Sjöström M. (2011). High folate intake is related to better academic achievement in swedish adolescents. Pediatrics.

[117] Petridou E.T., Kousoulis A.A., Michelakos T., Papathoma P., Dessypris N., Papadopoulos F.C., Stefanadis C. (2015). Folate and B12 serum levels in association with depression in the aged: A systematic review and meta-analysis. Aging Ment. Health.

[118] Michelakos T., Kousoulis A.A., Katsiardanis K., Dessypris N., Anastasiou A., Katsiardani K.-P., Kanavidis P., Stefanadis C., Papadopoulos F.C., Petridou E.T. (2013). Serum folate and B12 levels in association with cognitive impairment among seniors results from the velestino study in greece and meta-analysis. J. Aging Health.

[119] O’Leary F., Allman-Farinelli M., Samman S. (2012). Vitamin B12 status, cognitive decline and dementia: A systematic review of prospective cohort studies. Br. J. Nutr..

[120] Doets E.L., van Wijngaarden J.P., Szczecińska A., Dullemeijer C., Souverein O.W., Dhonukshe-Rutten R.A., Cavelaars A.E., van’t Veer P., Brzozowska A., de Groot L.C. (2013). Vitamin B12 intake and status and cognitive function in elderly people. Epidemiol. Rev..

[121] Lopes da Silva S., Vellas B., Elemans S., Luchsinger J., Kamphuis P., Yaffe K., Sijben J., Groenendijk M., Stijnen T. (2014). Plasma nutrient status of patients with alzheimer’s disease: Systematic review and meta-analysis. Alzheimer’s Dement..

[122] Malouf R., Grimley Evans J. (2008). Folic acid with or without vitamin B12 for the prevention and treatment of healthy elderly and demented people. Cochrane Database Syst. Rev..

[123] Malouf R., Areosa Sastre A. (2003). Vitamin B12 for cognition. Cochrane Database Syst. Rev..

[124] Malouf R., Grimley Evans J. (2003). Vitamin B6 for cognition. Cochrane Database Syst. Rev..

[125] Dangour A.D., Whitehouse P.J., Rafferty K., Mitchell S.A., Smith L., Hawkesworth S., Vellas B. (2010). B-vitamins and fatty acids in the prevention and treatment of alzheimer’s disease and dementia: A systematic review. J. Alzheimer’s Dis..

[126] Balk E.M., Raman G., Tatsioni A., Chung M., Lau J., Rosenberg I.H. (2007). Vitamin B6, B12, and folic acid supplementation and cognitive function: A systematic review of randomized trials. Arch. Intern. Med..

[127] Wald D.S., Kasturiratne A., Simmonds M. (2010). Effect of folic acid, with or without other B vitamins, on cognitive decline: Meta-analysis of randomized trials. Am. J. Med..

[128] Ford A.H., Almeida O.P. (2012). Effect of homocysteine lowering treatment on cognitive function: A systematic review and meta-analysis of randomized controlled trials. J. Alzheimer’s Dis..

[129] Garrard P., Jacoby R. (2015). B-vitamin trials meta-analysis: Less than meets the eye. Am. J. Clin. Nutr..

[130] Smith A.D., de Jager C.A., Refsum H., Rosenberg I.H. (2015). Homocysteine lowering, B vitamins, and cognitive aging. Am. J. Clin. Nutr..

[131] McCaddon A., Miller J.W. (2015). Assessing the association between homocysteine and cognition: Reflections on bradford hill, meta-analyses, and causality. Nutr. Rev..

[132] Durga J., van Boxtel M.P.J., Schouten E.G., Kok F.J., Jolles J., Katan M.B., Verhoef P. (2007). Effect of 3-year folic acid supplementation on cognitive function in older adults in the facit trial: A randomised, double blind, controlled trial. Lancet.

[133] Jager C.A., Oulhaj A., Jacoby R., Refsum H., Smith A.D. (2012). Cognitive and clinical outcomes of homocysteine-lowering B-vitamin treatment in mild cognitive impairment: A randomized controlled trial. Int. J. Geriatr. Psychiatry.

[134] Tangney C., Aggarwal N., Li H., Wilson R., Decarli C., Evans D., Morris M. (2011). Vitamin B12, cognition, and brain mri measures a cross-sectional examination. Neurology.

[135] Vogiatzoglou A., Refsum H., Johnston C., Smith S., Bradley K., de Jager C., Budge M., Smith A. (2008). Vitamin B12 status and rate of brain volume loss in community-dwelling elderly. Neurology.

[136] De Lau L., Smith A., Refsum H., Johnston C., Breteler M. (2009). Plasma vitamin B12 status and cerebral white-matter lesions. J. Neurol. Neurosurg. Psychiatry.

[137] Smith A.D., Smith S.M., de Jager C.A., Whitbread P., Johnston C., Agacinski G., Oulhaj A., Bradley K.M., Jacoby R., Refsum H. (2010). Homocysteine-lowering by b vitamins slows the rate of accelerated brain atrophy in mild cognitive impairment: A randomized controlled trial. PLoS ONE.

[138] Douaud G., Refsum H., de Jager C.A., Jacoby R., Nichols T.E., Smith S.M., Smith A.D. (2013). Preventing alzheimer’s disease-related gray matter atrophy by b-vitamin treatment. Proc. Natl. Acad. Sci. USA.

[139] Almeida O.P., Ford A.H., Flicker L. (2015). Systematic review and meta-analysis of randomized placebo-controlled trials of folate and vitamin B12 for depression. Int. Psychogeriatr..

[140] Strain J., Hughes C.F., McNulty H., Ward M. (2015). Riboflavin lowers blood pressure: A review of a novel gene-nutrient interaction. Nutr. Food Sci. Res..

[141] Singer G.M., Geohas J. (2006). The effect of chromium picolinate and biotin supplementation on glycemic control in poorly controlled patients with type 2 diabetes mellitus: A placebo-controlled, double-blinded, randomized trial. Diabetes Technol. Ther..

[142] Albarracin C., Fuqua B., Geohas J., Juturu V., Finch M.R., Komorowski J.R. (2007). Combination of chromium and biotin improves coronary risk factors in hypercholesterolemic type 2 diabetes mellitus: A placebo-controlled, double-blind randomized clinical trial. J. Cardiometab. Syndr..

[143] Fernandez-Mejia C., Zendejas-Ruiz I., Revilla-Monsalve C., Islas-Andrade S., Baez-Saldana A., Cardenas A., Rojas-Ochoa A. (2003). Biotin treatment increases insulin sensitivity in type 2 diabetics. Diabetes.

[144] Hemmati M., Babaei H., Abdolsalehei M. (2013). Survey of the effect of biotin on glycemic control and plasma lipid concentrations in type 1 diabetic patients in kermanshah in Iran (2008–2009). Oman Med. J..

[145] Fernandez-Mejia C. (2005). Pharmacological effects of biotin. J. Nutr. Biochem..

[146] Lavigne P.M., Karas R.H. (2013). The current state of niacin in cardiovascular disease prevention: A systematic review and meta-regression. J. Am. Coll. Cardiol..

[147] Kuvin J.T., Dave D.M., Sliney K.A., Mooney P., Patel A.R., Kimmelstiel C.D., Karas R.H. (2006). Effects of extended-release niacin on lipoprotein particle size, distribution, and inflammatory markers in patients with coronary artery disease. Am. J. Cardiol..

[148] Benton D., Griffiths R., Haller J. (1997). Thiamine supplementation mood and cognitive functioning. Psychopharmacology.

[149] Doshi S.N., McDowell I.F.W., Moat S.J., Payne N., Durrant H.J., Lewis M.J., Goodfellow J. (2002). Folic acid improves endothelial function in coronary artery disease via mechanisms largely independent of homocysteine lowering. Circulation.

[150] Obad A., Palada I., Valic Z., Ivancev V., Bakovic D., Wisloff U., Brubakk A.O., Dujic Z. (2007). The effects of acute oral antioxidants on diving-induced alterations in human cardiovascular function. J. Physiol. Lond..

[151] Katz D.L., Nawaz H., Boukhalil J., Giannamore V., Chan W., Ahmadi R., Sarrel P.M. (2001). Acute effects of oats and vitamin E on endothelial responses to ingested fat. Am. J. Prev. Med..

[152] Title L.M., Cummings P.M., Giddens K., Nassar B.A. (2000). Oral glucose loading acutely attenuates endothelium-dependent vasodilation in healthy adults without diabetes: An effect prevented by vitamins c and e. J. Am. Coll. Cardiol..

[153] Usui M., Matsuoka H., Miyazaki H., Ueda S., Okuda S., Imaizumi T. (1999). Endothelial dysfunction by acute hyperhomocyst (e) inaemia: Restoration by folic acid. Clin. Sci..

[154] Hartvig P., Lindner K., Bjurling P., Långström B., Tedroff J. (1995). Pyridoxine effect on synthesis rate of serotonin in the monkey brain measured with positron emission tomography. J. Neural Transm. Gen. Sect. JNT.

[155] Ebben M., Lequerica A., Spielman A. (2002). Effects of pyridoxine on dreaming: A preliminary study. Percept. Mot. Skills.

[156] Haskell C.F., Scholey A.B., Jackson P.A., Elliott J.M., Defeyter M.A., Greer J., Robertson B.C., Buchanan T., Tiplady B., Kennedy D.O. (2008). Cognitive and mood effects in healthy children during 12 weeks’ supplementation with multi-vitamin/minerals. Br. J. Nutr..

[157] Scholey A., Bauer I., Neale C., Savage K., Camfield D., White D., Maggini S., Pipingas A., Stough C., Hughes M. (2013). Acute effects of different multivitamin mineral preparations with and without guaraná on mood, cognitive performance and functional brain activation. Nutrients.

[158] White D.J., Camfield D.A., Maggini S., Pipingas A., Silberstein R., Stough C., Scholey A. (2014). The effect of a single dose of multivitamin and mineral combinations with and without guaraná on functional brain activity during a continuous performance task. Nutr. Neurosci..

[159] Kennedy D.O., Stevenson E., Jackson P., Wishart K., Bieri G., Barella L., Carne A., Dunn S., Robertson B., Forster J. (2016). Multivitamins/minerals modulate cerebral blood-flow and whole-body energy metabolism during cognitive tasks of graded difficulty. J. Nutr. Metab..

[160] Benton D. (2001). Micro-nutrient supplementation and the intelligence of children. Neurosci. Biobehav. Rev..

[161] Eilander A., Gera T., Sachdev H.S., Transler C., van der Knaap H.C.M., Kok F.J., Osendarp S.J.M. (2010). Multiple micronutrient supplementation for improving cognitive performance in children: Systematic review of randomized controlled trials. Am. J. Clin. Nutr..

[162] Frensham L.J., Bryan J., Parletta N. (2012). Influences of micronutrient and omega-3 fatty acid supplementation on cognition, learning, and behavior: Methodological considerations and implications for children and adolescents in developed societies. Nutr. Rev..

[163] Grima N.A., Pase M.P., Macpherson H., Pipingas A. (2012). The effects of multivitamins on cognitive performance: A systematic review and meta-analysis. J. Alzheimer’s Dis..

[164] White D.J., Cox K.H., Peters R., Pipingas A., Scholey A.B. (2015). Effects of four-week supplementation with a multi-vitamin/mineral preparation on mood and blood biomarkers in young adults: A randomised, double-blind, placebo-controlled trial. Nutrients.

[165] Haskell C.F., Robertson B., Jones E., Forster J., Jones R., Wilde A., Maggini S., Kennedy D.O. (2010). Effects of a multi-vitamin/mineral supplement on cognitive function and fatigue during extended multi-tasking. Hum. Psychopharmacol. Clin. Exp..

[166] Grodstein F., O’Brien J., Kang J.H., Dushkes R., Cook N.R., Okereke O., Manson J.E., Glynn R.J., Buring J.E., Gaziano J.M. (2013). Long-term multivitamin supplementation and cognitive function in mena randomized trial. Ann. Intern. Med..

[167] Harris E., Macpherson H., Vitetta L., Kirk J., Sali A., Pipingas A. (2012). Effects of a multivitamin, mineral and herbal supplement on cognition and blood biomarkers in older men: A randomised, placebo-controlled trial. Hum. Psychopharmacol. Clin. Exp..

[168] Macpherson H., Ellis K.A., Sali A., Pipingas A. (2012). Memory improvements in elderly women following 16 weeks treatment with a combined multivitamin, mineral and herbal supplement. Psychopharmacology.

[169] Harris E., Kirk J., Rowsell R., Vitetta L., Sali A., Scholey A.B., Pipingas A. (2011). The effect of multivitamin supplementation on mood and stress in healthy older men. Hum. Psychopharmacol. Clin. Exp..

[170] Pipingas A., Camfield D.A., Stough C., Scholey A.B., Cox K.H., White D., Sarris J., Sali A., Macpherson H. (2014). Effects of multivitamin, mineral and herbal supplement on cognition in younger adults and the contribution of B group vitamins. Hum. Psychopharmacol. Clin. Exp..

[171] Pipingas A., Camfield D., Stough C., Cox K., Fogg E., Tiplady B., Sarris J., White D., Sali A., Wetherell M. (2013). The effects of multivitamin supplementation on mood and general well-being in healthy young adults. A laboratory and at-home mobile phone assessment. Appetite.

